# C-Type Lectins from Marine Bivalves: Functional Diversity and Structural Insights

**DOI:** 10.3390/md24010017

**Published:** 2025-12-26

**Authors:** Ivan Buriak, Daria Lanskikh, Ivan Baklanov, Daniil Kozyrev, Andrei Grinchenko

**Affiliations:** A.V. Zhirmunsky National Scientific Center of Marine Biology, Far Eastern Branch, Russian Academy of Sciences, 690041 Vladivostok, Russia

**Keywords:** C-type lectins, bivalve mollusks, innate immunity, protein structure, carbohydrate specificity

## Abstract

C-type lectins (CTLs) are a large family of calcium-dependent carbohydrate-binding proteins that play crucial roles in innate immunity as pattern recognition receptors. Bivalve mollusks possess exceptionally diverse and expanded repertoires of CTLs, yet a systematic review integrating their structural, functional, and regulatory aspects has been lacking. This article provides a comprehensive synthesis of current knowledge on bivalve CTLs, analyzing their biosynthesis, complex tissue-specific expression under both normal and stressed conditions, and their multifaceted roles in immune defense and other physiological processes. Our analysis consolidates data on their diverse domain architectures, phylogenetic relationships, and the variability of key motifs within their carbohydrate-recognition domains. The results demonstrate that bivalve CTLs are not only critical for pathogen recognition, agglutination, and phagocytosis but also involved in processes like nutrition, development, byssus formation and biomineralization. However, a significant finding is that the detailed carbohydrate specificity for most bivalve CTLs remains poorly characterized, often limited to monosaccharide inhibition assays. In conclusion, while the immune role of bivalve CTLs is well-established, this review underscores a critical gap in understanding their fine glycan-binding profiles. Therefore, a shift in the focus of future research towards elucidating their structure and carbohydrate specificity is required for a full understanding of their biological functions and an assessment of their biomedical potential.

## 1. Introduction

C-type lectins (CTLs) are a large and structurally diverse family of carbohydrate-recognizing proteins that play key roles in a wide range of physiological processes, including homeostasis, innate and adaptive immunity. Historically, they were among the first animal lectin families to be discovered. Although the first observations of lectin activity in animals, such as the agglutination of red blood cells by rattlesnake venom, were made by S. Weir Mitchell in the 1860s, and proteins like conglutinin (1906) were described long ago, their nature as carbohydrate-binding proteins was recognized much later [1]. A turning point in the formation of the CTL concept came with research in the 1970s that led to the discovery and isolation of the hepatic asialoglycoprotein receptor (ASGPR). In 1988, Kurt Drickamer, after analyzing the accumulated structural data, proposed classifying Ca^2+^-dependent lectins, structurally homologous to ASGPR, into a separate group, C-type lectins [2]. This marked the beginning of a systematic study of the family, which now comprises thousands of members. A unifying feature of CTLs is the presence of a conserved carbohydrate-recognition domain (CRD), now more commonly referred to as a CTLD (C-type lectin-like domain), which is characterized by a specific structure of antiparallel β-sheets and α-helices stabilized by conserved disulfide bonds. Although carbohydrate binding in most CTLs depends on Ca^2+^ ions, which is mediated by sugar coordination by specific motifs (such as EPN or WND) within the CTLD, some proteins with this domain have lost the ability to bind ligands or calcium and have acquired other functions. CTLs family includes collectins, selectins, endocytic receptors, lecticans and some other (16 groups that are distinguished by CTLDs domain architecture), which function as pattern-recognition receptors (PRRs), adhesion receptors, and signaling molecules, demonstrating how an evolutionarily ancient structural scaffold has been adapted to perform a variety of biological functions [3,4,5].

Nevertheless, the primary function of CTLs, like other carbohydrate-binding proteins, is the recognition of glycocode, or sugar code [6,7]. This ability determines the enormous significance, biological role, and diversity of processes involving lectins, as well as their biomedical potential, including immunohematology for blood typing and cell surface analysis [8], histochemistry and cell biology for detecting glycans in cells and tissues [9], analytical chemistry for biosensing and chromatography [10,11], nanotechnology for targeted drug delivery systems [12], as well as promising diagnostic and therapeutic agents with antiviral [13,14], antimicrobial [15] and antitumor activity [16,17]. Moreover, the best-studied mammalian CTLs, in addition to canonical carbohydrate binding [7], have also been shown to interact with other ligands via various parts of their CTLD [18,19], further expanding their potential role and application. Invertebrate CTLs have been studied primarily as immune molecules capable of recognizing carbohydrate motifs of pathogen-associated molecular patterns (PAMPs), thus serving as pattern-recognition receptors in both soluble and membrane-bound forms [20,21,22,23]. However, information regarding the spectrum of their carbohydrate specificity is extremely limited and fragmentary.

On the other hand, protein functionality is determined by its structure. In recent years, there has been a shift in understanding the mechanisms of lectin-carbohydrate interactions, moving from simple point intermolecular contact to more complex surface interactions [24]. Furthermore, with the development of molecular modeling and genetic engineering methods, the question of the spatial organization of lectins and their complexes with ligands has become even more pressing, as it is necessary both for the verification of new protein models and the effective rational design of existing ones [25,26]. Due to their long history of study and widespread occurrence in nature, a number of well-studied high-resolution CTLD structures now exist, most of which pertain to vertebrates and, to a lesser extent, invertebrates. Meanwhile, the development of next-generation sequencing (NGS) and proteomics technologies in recent decades has revealed the presence of a vast number of CTLs in invertebrates. Bivalves are particularly notable in this regard [27], but a systematic review of their CTLs has not been conducted in recent years, and this is the focus of this work.

## 2. Biosynthesis and Tissue Distribution

A hallmark feature of CTLs in bivalves is not only their substantial gene family expansion [28,29] and structural diversity [27,29] but also their complex, tissue-specific expression patterns, which are dynamically regulated in response to a wide array of biotic and abiotic factors. This section summarizes current knowledge on CTLs expression profiles across different tissues and their regulation upon exposure to pathogens and environmental stressors.

Data on the tissue distribution of CTLs in bivalves have been primarily obtained using quantitative real-time PCR (qPCR) and, to a lesser extent, by immunohistochemistry (IHC), in situ hybridization (ISH), semi-quantitative PCR, and RNA sequencing. Expression analyses reveal their constitutive presence in a broad range of tissues, most commonly including hemocytes, the hepatopancreas, muscle, mantle, gills, and gonads.

### 2.1. Tissue-Specific Expression of C-Type Lectins

The constitutive expression patterns of CTLs are closely linked to their functional specialization. CTLs play a pivotal role in invertebrate innate immunity. They act as PRRs that bind to PAMPs such as lipopolysaccharide (LPS), peptidoglycan (PGN), glucan, mannan and poly I:C [28], facilitating microbial clearance through interactions with immune cell receptors, direct lysis, or opsonization [27]. Furthermore, some CTLs can activate the complement system [30]. Consistent with these immune functions, expression analysis reveals that CTLs are predominantly expressed in immunocompetent tissues and organs directly exposed to the environment. The highest levels of constitutive expression are typically detected in the hepatopancreas, gills, mantle, and hemocytes (Table 1).

The hepatopancreas stands out as a key site of CTLs expression, with transcript levels for some CTLs exceeding those in hemocytes by 500-fold or more [33,34,36]. This is particularly evident for CTLs from the following species: *A. irradians* (AiCTL-3, AiCTL-6, AiCTL-7, AiCTL-9, AiLe) [33,34,35,36,37], *M. gigas* (CgCLec-1, CgCLec-4, CgLec-4E) [39,42,45], *R. philippinarum* (RpCTL, VpClec-1, VpClec-2, VpClec-3, VpClec-4, VpCTL, CTL-2, CTL-3, CTL-4, CTL-5, CTL-6) [53,54,55,57], and *S. constricta* (ScCTL, ScCTL-1) [67,68]. The overwhelming majority of these CTLs have experimentally demonstrated abilities to bind specific PAMPs, exhibit antibacterial and agglutinating activity, and possess pronounced opsonic properties. This broad functional repertoire underscores the critical contribution of the hepatopancreas to both humoral and cellular immunity in invertebrates.

Certain CTLs show higher representation in the gills [44,58] and mantle [40,41,49,51]. This tissue specificity points to a paramount role for these lectins in the immune defense of mucosal barriers. Proteomic analysis has identified the presence of approximately 10 CTLs in the mucus secreted by the pallial organs (gills, mantle, labial palps) of the oyster *C. virginica*, confirming their key function as soluble PRRs in the first line of defense at the host-environment interface [72].

A number of lectins from *M. gigas* (CgCLec-5, CgCLec-CCP, CgCLEC-TM2) [30,42,43], *R. philippinarum* (VpCTL) [56], *A. irradians* (AiCTL1) [32], and *M. crassicostata* (Cnlec-1) [48] exhibit their highest expression levels in hemocytes compared to other investigated tissues. Some of these function on the cell membrane. CgCLEC-TM2, for instance, possesses a transmembrane domain and appears to regulate downstream immune cascades by influencing the expression of interleukin-17 (CgIL17-1 and CgIL17-4) in oyster hemocytes.

Species and isoform diversity is a hallmark feature of the CTL family. Within the same species, different isoforms often exhibit unique, non-overlapping expression patterns. In the Manila clam *R. philippinarum*, isoforms CTL-1 and CTL-5 are most highly expressed in the gills, while CTL-2, CTL-4, and CTL-6 show peak expression in the hepatopancreas, and CTL-3 is detected at very low levels across all tissues [57]. Similarly, among the five lectins SsCTL1–SsCTL5 characterized in the clam *A. kagoshimensis*, tissue profiles vary considerably, ranging from ubiquitous expression (SsCTL4, SsCTL5) to a profile strictly restricted to the hepatopancreas (SsCTL2) [31]. This diversity underscores the profound functional specialization within the CTL family, which facilitates a broad recognition repertoire.

### 2.2. Regulation of C-Type Lectin Expression During Immune Response

Although basal expression patterns are highly species-specific and unique to each lectin studied, a key feature is their capacity for powerful induction upon immune challenge. This is most pronounced in hemocytes, where even initially low transcript levels can be significantly upregulated following stimulation with pathogens or their molecular patterns. A similar trend is observed in other tissues, such as the hepatopancreas, gills, and mantle [31,55,67]. For instance, immunohistochemical analysis confirmed MCL3 expression in *R. philippinarum* infected with *Perkinsus olseni* across all these tissues—the digestive gland, gills, mantle, and intestine [52]—highlighting their active role in mounting both systemic and mucosal immune responses.

The response to bacterial infections is characterized by rapid and often robust transcriptional induction. In the scallop *S. farreri*, infection with *Listonella anguillarum* caused a 328-fold increase in Cflec-3 expression in hemocytes within 12 h [61]. The strength and kinetics of the response can vary depending on the bacterial species (Table 2). For example, expression of AiCTL-9 in *A. irradians* is activated in response to *Vibrio anguillarum* and *Pichia pastoris*, but weakly by *Micrococcus luteus* [36]. Activation of CFLec-1 in *S. farreri* [58], and AiCTL-3 and AiCTL-6 in *A. irradians* [33,34] has also been noted in response to both Gram-positive and Gram-negative bacteria. Expression of AiLec in the hemocytes of *A. irradians* is also activated by Gram-positive and Gram-negative bacteria, but not by fungi [37].

Downregulation of a small number of CTLs in response to pathogens has also been described. For instance, transcript levels of SsCTL5 in *A. kagoshimensis* significantly decrease after exposure to *V. parahaemolyticus* [31]. Reduced levels of CvML3914 in *C. virginica* in response to *V. alginolyticus* [73] and CfLec-1 in *S. farreri* in response to PGN have also been observed [59].

The response to PAMPs also demonstrates high specificity. A transcriptome analysis of *M. gigas* revealed that out of 299 CTL genes, 80 were upregulated by at least one of four tested PAMPs: LPS, PGN, glucan, and poly(I:C). A pronounced response specificity was observed, with distinct sets of CTL genes responding to different stimuli: 32 CTLs were activated by LPS, 29 by PGN, 32 by glucan, and 47 by poly(I:C). Only four CTL genes exhibited a universal response to all four stimulation types, all of which were secreted forms lacking transmembrane domains [28]. This differential expression pattern in response to distinct PAMPs has also been reported by other authors for *M. gigas* and *S. farreri* [59,65,74]. Examples of CTLs whose expression is universally upregulated in hemocytes by various PAMPs include CfLec-2 and CfLec-4 in *S. farreri* [60,63].

Furthermore, there is evidence that CTLs may contribute to immune priming. Upon re-infection of immunized *S. farreri*, an earlier and significantly stronger increase in the expression of five CTL isoforms (Cflec-1–Cflec-5) was observed in hemocytes. This enhanced response was largely specific to the bacterial species used for immunization (*V. anguillarum*), while challenge with a different pathogen (*M. luteus*) induced a markedly weaker reaction [75].

The individual expression profile of specific CTL isoforms can determine pathogen resistance. In *R. philippinarum* infected with *V. anguillarum*, the expression level of the gene *evm.TU.xfSc0000193.14* was significantly higher in resistant individuals than in susceptible ones. Conversely, another isoform (*evm.TU.xfSc0000495.7*) showed the opposite dynamic [76]. The increased CTL expression in resistant clams in this case was associated with the activation of phagocytosis, likely underpinning their enhanced resistance to bacterial infection.

Algal biotoxins can differentially affect CTL expression dynamics. For example, activation of the CTL-A subfamily in *Mytilus galloprovincialis* was shown upon exposure to toxic algae (*Alexandrium minutum*, *Pseudo-nitzschia australis*) in the hepatopancreas [77]. A similar response to the diatom toxin from *Pseudo-nitzschia* was identified in the digestive gland of the scallop *Aequipecten opercularis* [77]. In contrast, transcriptomic analysis of *A. irradians* gills after 48 h exposure to okadaic acid (produced by *Dinophysis* and *Prorocentrum* species) revealed suppression of genes encoding CTLs [78].

A complex, biphasic expression pattern of CTL genes in the kidney of *M. yessoensis* was observed in response to the dinoflagellate *Alexandrium catenella* (which produces paralytic toxins): suppression on day 3 and activation on day 10 [79]. The sets of regulated genes in both phases showed little overlap. A similar dynamic was identified in *Mytilus chilensis* in response to saxitoxin (produced by various dinoflagellate species) [80]. These findings suggest a biphasic immune strategy in some bivalves: an initial suppression of immune functions is followed by compensatory activation of CTLs for long-term defense under chronic stress conditions.

### 2.3. Influence of Abiotic Stressors on C-Type Lectin

CTL expression serves as a sensitive indicator of physiological stress caused by environmental factors linking immune function to overall organismal resilience.

Thermal stress exerts complex effects on CTL expression across mollusk species. Low-temperature stress (−2 °C) in *R. philippinarum* caused significant induction of six CTL isoforms in the hepatopancreas [57]. Conversely, exposure to high temperatures (28 °C) in the scallop *S. farreri* suppressed CfLec-1 expression in hemocytes [81]. Injection of a β-adrenergic receptor antagonist during heat stress significantly increased CfLec-1 mRNA levels, indicating that catecholamines released during thermal stress complexly modulate the immune response via adrenergic receptors [81]. Combined exposure to high temperature and bacterial infection caused the strongest induction of CTLs in the clam *Meretrix petechialis*, which correlated with increased mortality [82].

CTL gene expression changes dynamically in response to air exposure, with temperature being a critical factor modulating the immune response. Expression of RpCTL in the gills of *R. philippinarum* increased rapidly during air exposure at both high (28 °C) and low (4 °C) temperatures. The response in the hepatopancreas was more complex: expression increased under low-temperature stress but decreased under high-temperature stress. Air exposure at high temperature caused a stronger and more acute regulation of RpCTL compared to low-temperature exposure. This correlated with the physiological condition of the clams: while all individuals survived low-temperature stress, high-temperature exposure led to significant mortality [83].

Ocean acidification (elevated pCO_2_) can also disrupt normal immune regulation in mollusks. In the mussel *M. chilensis*, exposure to 1200 µatm pCO_2_ led to an initial increase in CTL expression in the gills; however, subsequent infection with *V. anguillarum* did not induce but sharply suppressed it [84]. This indicates that acidification renders the immune system incapable of mounting an adequate response to bacterial infection.

Other environmental parameters also influence CTL expression. For instance, hypoxic stress suppresses the expression of a CTL gene in *C. virginica* [85]. In contrast, reduced salinity caused a statistically significant increase in CTL expression levels in *A. kagoshimensis* [86].

Pollutants generally exert an immunosuppressive effect. Copper exposure suppressed the expression of 16 CTL transcripts in the gills of the scallop *M. yessoensis* [87]. The immunotoxicant BPDE and the neuroendocrine factor norepinephrine suppressed the expression of lectins Lec-1 and Lec-2 in the hemocytes of the scallop *S. farreri* [88]. A study on the effects of the xenoestrogen 4-nonylphenol on the immune function of the oyster *M. gigas* revealed a selective influence on CTL expression. Exposure to a low dose of the toxicant caused a decrease in Lec2 transcript levels, whereas a high dose had no statistically significant effect on the expression of this gene [89].

It is noteworthy that beyond pathogen defense, some CTLs play roles in developmental processes. Immune competence in bivalves forms during early ontogeny, as evidenced by the expression of CTL genes. In the Pacific oyster *M. gigas*, C-type lectin 3 shows a peak expression at the embryonic, trochophore, and D-larva stages, localizing to the velum. Infection with *V. splendidus* enhances immunoreactivity, confirming the early establishment of a functional immune response [90]. In the Japanese scallop *M. yessoensis*, the ontogenetic profile of lectin MyCLF shows its activation at the trochophore stage and a sharp increase in expression during metamorphosis [49]. A similar pattern is observed in the clam *L. sieboldii*, where significant activation of CTL genes also occurs at metamorphosis, suggesting their involvement in cell adhesion and tissue remodeling during this critical transformation period [91].

Furthermore, mucosal CTLs (such as CvML and MeML) are critical for the selective capture of food particles [66], and their expression is sharply induced upon starvation, confirming their role in nutrition [38,50,73].

In summary, the complex and dynamic expression patterns of CTLs underpin the immune plasticity of bivalve mollusks. Their ability to fine-tune expression in response to specific biotic factors and abiotic stress (temperature, pollutants, acidification) makes CTLs a key element in maintaining homeostasis in a variable marine environment.

## 3. Antibacterial Properties and Immune Functions

In the previous chapter, the tissue distribution of CTLs in marine invertebrates and their biosynthesis at basal expression levels, as well as under bacterial infection or PAMP injection, were described. This chapter provides a more detailed examination of the functions of invertebrate CTLs in immunological defense, specifically their roles in recognizing and binding various PAMPs, bacterial agglutination, and the enhancement of hemocyte phagocytosis and encapsulation (see Table 3).

Studies on the Manila clam *R. philippinarum* have revealed numerous CTLs with diverse immune functions. The lectins VpClec-1 and VpClec-2 exhibited different PAMP-binding capacities: recombinant VpClec-1 bound LPS, PGN, glucan, and zymosan, whereas VpClec-2 bound only LPS, glucan, and zymosan. Both exhibited broad antibacterial activity against *V. harveyi*, *V. splendidus*, *V. anguillarum*, *E. cloacae*, and *A. hydrophila*, and enhanced hemocyte phagocytosis and encapsulation; notably, VpClec-1 also induced hemocyte chemotaxis [54]. Two other lectins, VpClec-3 and VpClec-4, also exhibited broad agglutinating activity against *V. harveyi*, *V. splendidus*, and *V. anguillarum*; however, VpClec-3 did not agglutinate *E. cloacae* and *A. hydrophila*. They displayed distinct PAMP-binding profiles: rVpClec-4 bound LPS and glucan, while rVpClec-3 bound only PGN. Both proteins enhanced hemocyte phagocytosis [55]. The lectin VpCTL displayed a broad agglutination spectrum against Gram-positive bacteria (*S. aureus*), Gram-negative bacteria (*E. coli*, *V. parahaemolyticus*, *V. harveyi*, *P. putida*, *P. mirabilis*), and fungi (*P. pastoris*) [96]. VpCTL significantly enhanced phagocytic and encapsulating activity of hemocytes. Its mRNA expression was strongly upregulated in hemocytes following *L. anguillarum* challenge, indicating its role in the immune response [56]. RpCTL showed antimicrobial activity by inhibiting growth of *S. aureus*, *B. subtilis*, *E. coli*, and *V. anguillarum*. Administration of recombinant RpCTL significantly increased clam survival after infection with *V. anguillarum*, demonstrating the lectin’s direct protective function [53].

Several multifunctional CTLs have been characterized in the Pacific oyster *M. gigas*. The lectin CgCLEC-TM2 exhibited Ca^2+^-dependent agglutination against a broad range of microorganisms, including *V. anguillarum*, *B. subtilis*, *V. splendidus*, *E. coli*, *P. pastoris*, *S. aureus*, and *M. luteus*. Its expression in hemocytes was induced by *V. splendidus*, and knockdown led to reduced phagocytosis and decreased interleukin-17 (CgIL17) levels, indicating its role in pathogen recognition and immune response regulation [43]. The lectin CgCLec, containing a CCP domain, participates in complement activation by interacting with the serine protease CgMASP1-1 to cleave the C3 complement component, modulating processes such as inflammation, phagocytosis, chemotaxis, and cell lysis. Knockdown of CgCLec-CCP resulted in reduced phagocytosis, cytokine production, and antimicrobial peptides. Recombinant rCgCLec-CCP bound *E. coli*, *V. splendidus*, *S. aureus*, *M. luteus*, as well as LPS and PGN [30]. CgCLec-2 bound various PAMPs (LPS, mannan, PGN), showed strong binding affinity for *V. anguillarum*, *V. splendidus*, and *Y. lipolytica*, enhanced phagocytosis, and inhibited *S. aureus* growth. It has also been suggested to potentially participate in complement activation [40]. CgCLec-3 bound LPS and PGN, demonstrated strong binding capacities for *V. anguillarum* and *V. splendidus*, agglutinated microorganisms in a Ca^2+^-dependent manner, enhanced phagocytosis, and exhibited direct antibacterial activity against *E. coli* and *S. aureus*, indicating its function as both a PRR and immune effector [41]. CgLec-4E agglutinated and inhibited growth of *V. alginolyticus*, and its expression increased after *V. alginolyticus* infection, suggesting a role in mucosal immunity [45]. Two other lectins, CgCLec-4 and CgCLec-5, displayed different affinities for PAMPs (LPS, PGN, β-glucan, mannan) and microbes (*S. aureus*, *E. coli*, *V. anguillarum*, *Y. lipolytica*); rCgCLec-4 showed stronger agglutination and wider growth inhibition activity, while rCgCLec-5 exhibited higher PAMP-binding activity, illustrating functional diversification within the lectin family [42]. The receptor CgCLec-HTM binds LPS and various bacteria; by virtue of its hemITAM motif, upon ligand binding, it transduces signals via the intracellular CgSyk-ERK-Rel pathway, inducing production of CgIL-17 and CgTNF, as well as phagocytosis and cytotoxicity [95].

A considerable number of CTLs with diverse functions have been identified in the scallops *A. irradians* and *S. farreri*. Expression of AiCTL1 could be induced in response to injury and bacterial injection, suggesting roles in wound healing and immune response [32]. AiCTL-3, containing an EPN motif, bound LPS, PGN, glucan (but not mannan), and enhanced phagocytosis, functioning as a PRR and opsonin. Its mRNA expression was significantly upregulated after infection with *V. anguillarum* and *M. luteus* [33]. AiCTL5 agglutinated Gram-negative bacteria (*E. coli*, *L. anguillarum*) and rabbit erythrocytes but not Gram-positive bacteria (*Bacillus thuringiensis*, *M. luteus*), indicating specificity for LPS [92]. AiCTL-6 agglutinated both Gram-negative (*E. coli*) and Gram-positive (*M. luteus*, *S. aureus*) bacteria, and its mRNA expression was significantly elevated after challenge with *L. anguillarum* and *M. luteus* [34]. AiCTL-7 (containing EPD/WSD motifs) bound a wide spectrum of PAMPs (PGN, LPS, mannan, yeast glucan, poly(I:C)) and microbes (*S. aureus*, *E. coli*, *V. anguillarum*, *P. pastoris*, *Y. lipolytica*), and inhibited the growth of *E. coli*. Its structure suggests it may function similarly to collectins and selectins in the scallop’s immune defense [93]. The lectin also agglutinated *P. pastoris* in a Ca^2+^- and mannose-dependent manner but showed no activity against *L. ansguillarum* [35]. AiCTL-9, containing four CRDs, bound various PAMPs (LPS, PGN, mannan, glucan) and agglutinated *P. pastoris*, *B. subtilis*, *E. coli*, *and V. anguillarum* in a Ca^2+^-dependent manner; it also enhanced hemocyte adhesion and encapsulation [36].

In the scallop *S. farreri*, the lectin CFLec-1 bound LPS, PGN, and mannan; it agglutinated *E. coli* in a Ca^2+^-dependent manner (but not *M. luteus* or erythrocytes), and inhibited growth of *E. coli* and *M. luteus*, functioning as both a constitutive and inducible PRR [58]. Stimulation of scallops with typical PAMPs increased other C-type lectin with the same name CfLec-1 expression in response to LPS and β-glucan but significantly decreased it upon PGN stimulation. This lectin significantly enhanced the phagocytic activity of hemocytes and their encapsulation [59]. CfLec-2 bound LPS, PGN, mannan, and zymosan, but not glucan, and initiated hemocyte adhesion and encapsulation, combining pathogen recognition and adhesion functions. Its expression was significantly upregulated in hemocytes following stimulation with LPS, PGN, or β-glucan [60]. Recombinant CfLec-2 exhibited Ca^2+^-independent agglutination of *Staphylococcus haemolyticus*, inhibited by D-mannose, and suppressed growth of *E. coli* TOP10F′ [98]. CfLec-3 bound various PAMPs (LPS, PGN, yeast glucan, mannan) and microorganisms (*E. coli*, *V. anguillarum*, *S. aureus*, *P. pastoris*), mediating hemocyte phagocytosis and encapsulation against *E. coli*. CfLec-3 mRNA expression in hemocytes was significantly increased after stimulation with LPS, PGN, or β-glucan, but not poly(I:C) [62]. Its expression was also significantly upregulated after *L. anguillarum* infection. Ca^2+^- and mannose-dependent agglutination of *P. stutzeri* was observed [61]. CfLec-4, containing four CRDs, bound a broad spectrum of PAMPs (LPS, PGN, glucan, mannose) and microbes (*S. aureus*, *M. luteus*, *E. coli*, *V. anguillarum*, *P. pastoris*), and enhanced phagocytosis. Its expression was significantly elevated after stimulation with β-glucan, LPS, or PGN [63]. Functional analyses of individual CRDs showed that CRD1 and CRD2 bound LPS and mannan, while CRD3 and CRD4 possessed broader specificity (LPS, PGN, mannan, glucan) and opsonic activity, related to variability in their Ca^2+^-binding sites [97]. CfLec-5 agglutinated *P. pastoris* in a Ca^2+^-independent manner; its activity was inhibited by D-mannose, LPS, and glucan, but not by D-galactose or PGN [65]. Immunization of scallops with inactivated *V. anguillarum* induced an enhanced response of most of the above-mentioned CTLs upon re-exposure to the pathogen, indicating a phenomenon of immune priming [75].

Studies on other bivalve species further underscore the critical role of CTLs in immunity. In the mollusk *S. constricta*, the lectin ScCTL agglutinated *M. luteus*, *E. coli*, *V. anguillarum*, and *V. harveyi*, with its expression increasing after infection and during feeding, suggesting dual roles in immunity and nutrition [67]. ScCL exhibits Ca^2+^-independent activity and broad specificity, agglutinating not only pathogenic bacteria (*S. aureus*, *V. harveyi*) but also microalgae (most strongly *Chlorella vulgaris*), indicating its role in food recognition. ScCL shows high affinity for LPS and mannose [66]. ScCTL-1 exhibited higher binding specificity for *V. anguillarum* compared to *S. aureus*, whereas ScCTL-2 specifically bound and agglutinated Gram-negative bacteria (*E. coli*, *V. anguillarum*, *V. parahaemolyticus*) in a Ca^2+^-independent manner [68,69]. Expression of both lectins was significantly upregulated following bacterial challenge.

The lectin MmCTL4 from *M. meretrix* agglutinated *E. coli*, *B. subtilis*, and *S. aureus*, and its activity was inhibited by D-mannose, D-xylose, D-lactose, maltose, and LPS [46]. MmCTL5 also agglutinated pathogenic vibrios and bound various carbohydrates (glucose, D-mannose, D-galactose, maltose) [47]. Bacterial stimulation significantly increased expression of both lectins.

PmCTL-1 from *P. martensii* inhibited growth of Gram-positive bacteria (*M. luteus*, *S. aureus*, *B. subtilis*), but not Gram-negative bacteria [51]. In studies on the Japanese scallop *M. yessoensis*, the lectin MyCLF showed a rapid increase in expression after infection with *V. anguillarum* [49]. In the blood clam *T. granosa*, TgCTL-1 is an inducible secretory protein. The lectin demonstrated Ca^2+^-dependent agglutination and binding activity against *B. subtilis*, *S. aureus*, *E. coli*, *V. parahaemolyticus*, and *A. hydrophila*, and enhanced phagocytosis, functioning as an acute-phase protein [71]. The lectin GYL from *G. yessoensis* bound Gram-positive bacteria (*B. subtilis*, *S. aureus*), Gram-negative bacteria (*E. coli*, *V. proteolyticus*), and various PAMPs (PGN, LPS, β-1,3-glucan, mannan). Its expression could be induced by bacterial infection and environmental stress [94]. In the mollusk *S. grandis*, expression of SgCTL-1 was induced by LPS, PGN, and β-1,3-glucan, indicating its role as a PRR [70].

A novel putative C-type lectin (CvML) was identified in the Eastern oyster *C. virginica*; its expression was significantly upregulated after starvation and bacterial infection [38].

CTLs in marine bivalves are important participants in innate immunity, performing functions including pathogen recognition, agglutination, opsonization, enhancement of phagocytosis, activation of the complement cascade, and modulation of cell adhesion and inflammatory responses. Their ability to specifically bind diverse PAMPs underpins a broad spectrum of antimicrobial activity, making them promising targets for biomedical research.

## 4. Other Functions of C-Type Lectins

In addition to the immune role, several authors have noted a range of other functions, briefly mentioned in previous sections. CTLs also play a fundamental role in feeding and food particle capture, acting as key recognition receptors in the mucus and epithelia covering the pallial organs of bivalves. In the oyster *C. virginica*, two mucosal lectins CvML3912 and CvML3914 were identified, which are abundant in the mucus of the gills and labial palps. Suppression of their gene expression led to a significant reduction in the oyster’s ability to sort the microalgae *Dunaliella salina* and *Prasinocladus marinus* [73]. The regulation of mucosal CTLs expression in response to food stimuli has also been demonstrated. In the blue mussel *M. edulis*, the lectin MeML, expressed in mucocytes of the gills and labial palps, showed a 768-fold increase in expression in the labial palps and a 1207-fold increase in the gills after 5 days of starvation [50]. A similar pattern was observed in oysters, where starvation caused a 118-fold increase in CvML3912 expression and an 18-fold increase in CvML3914 expression [73]. In *S. constricta*, the expression of the ScCL lectin in the gills increased significantly within just 1–3 h after feeding with the microalgae *C. vulgaris* and *Thalassiosira pseudonana* [66], indicating rapid regulation of these molecules in response to food availability. Furthermore, CvML was also detected in mucocytes lining the epithelium of the digestive gland and pallial organs of *C. virginica* [38]. It is presumed that particles forming strong bonds with mucus lectins are directed to the dorsal tract of the gills for subsequent ingestion, while particles without such bonds are expelled through the ventral tract as pseudofeces [73].

An interesting example of CTLs multifunctionality is provided by six lectins (CTL-1–CTL-6) from the Manila clam *R. philippinarum*, which exhibit a conserved structure, including the presence of carbohydrate-recognition domains (CRDs) with characteristic EPN and WND motifs involved in carbohydrate binding [57]. The gene expression of these CTLs in the hepatopancreas and gills, which are the first lines of defense against pathogens, increases significantly under low-temperature stress. Transcripts of CTL-1 and CTL-2 show significant up-regulation over 12–96 h after exposure to a temperature of −2 °C, indicating their induction by cold. Similarly, CTL-4, CTL-5, and CTL-6 also show increased expression at various time points, indicating their involvement in the physiological processes of the organism’s response to cold. The authors cite several examples of similar CTLs expression reactions in other animals, as well as herring type II antifreeze protein (AFP), which, according to analysis of other studies, evolved from the carbohydrate-binding site of a CTL [99].

Another function, largely related to immunity, is cell adhesion. In vitro adhesion analysis using recombinant protein-coated agarose beads showed extremely high encapsulation of the beads by hemocytes for CfLec-2 from *S. farreri* and AiCTL-9 from *A. irradians* compared to the control: up to 97% of beads coated with rAiCTL-9 and 76% coated with rCfLec-2 were encapsulated within just 6 h of incubation, increasing to 87% for rCfLec-2 after 24 h [36,60]. In a study dedicated to the transcriptomic analysis of *Lutraria sieboldii* larvae, direct cell adhesion experiments were not conducted. The research focused on the transcriptomic profile during larval attachment and metamorphosis. As a result of KEGG analysis, the C-type lectin receptor signaling pathway was significantly enriched among the differentially expressed genes (DEGs) in the key module (turquoise module) associated with attachment. The authors suggest that CTLs may mediate adhesion to a specific substrate by recognizing suitable glycans on it and subsequently activating intracellular signaling (presumably via Syk and MAPK), which may promote degradation of the extracellular matrix (ECM) via matrix metalloproteinases (MMPs), a process characteristic of the morphogenesis of attachment structures such as the byssus gland in bivalve mollusks [91].

Furthermore, the involvement of CTLs in the formation of byssal structures has been demonstrated in *Atrina pectinata*. In particular, the key role of the protein apfp-1 (Atrina pectinata foot protein-1) in reducing mechanical stress in the proximal part of the byssus has been shown [100]. This part is embedded in soft tissues, creating a zone of mechanical mismatch (stiff-soft interface), and there is an abundant presence of other CTLs there, creating a sugar–lectin rich interface, presumably for the same purpose of protection against potential damage, ensuring the system’s durability under mechanical load [101].

**Figure 1:**
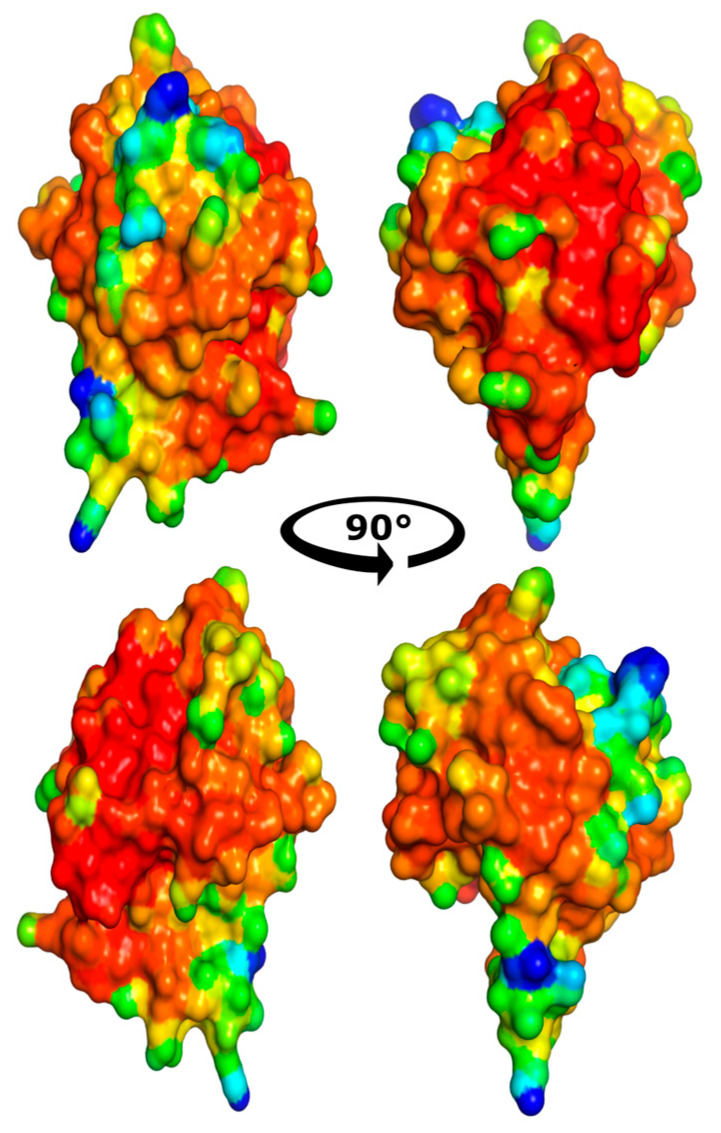
Model of surface electrostatic potential distribution on the carbohydrate-binding domain of CTL HcLecI (red surface—negatively charged regions of CTL HcLecI).

Another example of the structural role of CTLs is their involvement in the process of biomineralization. Specifically, they act as critically important structural elements by binding to chitin, the main polysaccharide of the organic matrix. Acting as a molecular bridge, they integrate the chitin scaffold with other proteins of the extracellular matrix, thereby forming an ordered three-dimensional structure that serves as a template for subsequent crystallization [102,103]. Beyond a passive structural role, CTLs actively regulate the very process of calcium carbonate crystallization. They can accelerate crystal nucleation, influence their precipitation rate, and directionally alter their morphology, promoting, for example, the fusion of small calcite crystals into larger, more complex aggregates. An additional level of regulation is provided by low-complexity regions enriched in serine and threonine, which, when phosphorylated, acquire a significant negative charge. This turns CTLs into effective “traps” for calcium ions, locally increasing their concentration in the mineralization zone and thereby catalyzing the formation of the mineral phase [103]. Moreover, the immune activity of HcLecI in the pearl mussel *Hyriopsis cumingii* proves to be critically important in situations combining damage and mineralization, such as shell repair after injury or the formation of the pearl sac around an implanted nucleus. In these scenarios, the same protein participates sequentially or in parallel in warding off potential infection and in organizing the new mineralized layer, demonstrating deep functional integration [104]. Our modeling data predict a structural feature of HcLecI CTL, namely the presence of extensive patches and pockets with negatively charged amino acids, which may promote calcium ion concentration (Figure 1). More about modeling pipelines is in Section 5. Thus, CTLs are not merely one component of the organic matrix but key regulatory nodes that ensure the structural integrity of the shell, directly control the kinetics and morphology of crystals, and serve as a molecular link between the systems of biomineralization and innate immunity, underscoring their evolutionary significance and adaptability in bivalve mollusks.

## 5. C-Type Lectins’ Structures

**Figure 2:**
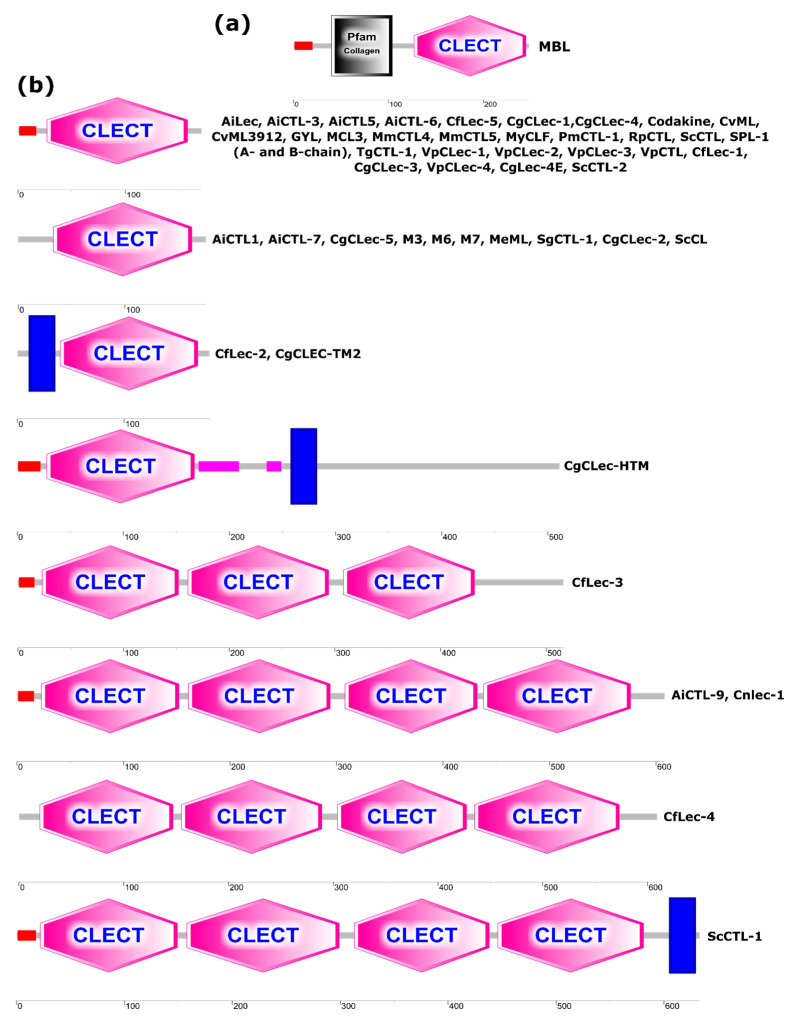
Domain organization of CTLs from bivalves. (**a**) Common domain architecture of CTLs from vertebrates; (**b**) common domain architecture of CTLs from bivalves. Red rectangle—signal peptide, pink rectangle—CTLD, blue rectangle—transmembrane region.

The proteins sequences from original articles with studied properties were used for bioinformatics analysis. The domain organization of bivalve CTLs is shown in Figure 2. The domain structure of vertebrate CTLs, using human MBL lectin as an example (Figure 2a), was used as a reference. Most of the bivalve lectins examined had a single-domain structure with or without a signal peptide. Among the single-domain CTLs, there are proteins with a transmembrane domain at the N-terminus of the amino acid sequence (CfLec-2, CgCLEC-TM2). Interestingly, a CTL with a unique C-terminal location of the transmembrane domain (CgCLec-HTM) was also found among them. Secreted and membrane-bound multidomain proteins are also found among CTLs. Thus, the secreted lectin CfLec-3 consists of three functional domains, while the secreted lectins AiCTL-9 and Cnlec-1 each consist of four domains. The lectin CfLec-4 was characterized as a protein consisting of four C-type domains but lacking a signal peptide. Of particular note is the unique structure of the membrane protein ScCTL-1, which contains a transmembrane domain at the C-terminus. The domain organization of CTLs in bivalves is summarized in Figure 2b. The variability of the domain architecture suggests that the evolution of CTLs in bivalves has led to significant complexity and variation in their molecular structure. This diversity reflects the adaptation of the invertebrate immune system to a wide range of pathogens and environmental conditions, requiring a greater diversity of receptors and effector molecules than in vertebrates. Domain structure annotation of CTLs from bivalves was performed using SMART (v10) in “Normal mode” with an additional search for Pfam domains, signal peptides and internal repeats using the default threshold [105].

**Figure 3:**
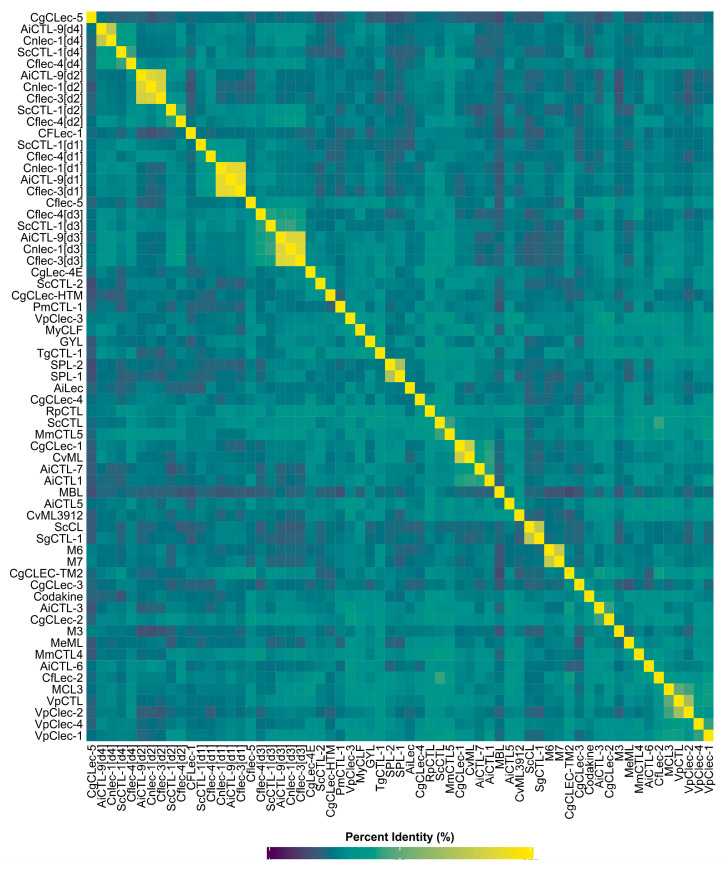
The percent identity matrix for CRD regions of CTLs from bivalves.

The percent identity matrix (PIM) (Figure 3) demonstrates extremely high variability in the amino acid sequences of the CRD domains of CTLs in bivalves. PIM analysis reveals distinct clusters of very high identity (>85%) between domains belonging to the same lectin. This is particularly noticeable in the lectin AiCTL-9 from *A. irradians*, whose d1, d2, d3, and d4 domains exhibit the expected low identity with each other (23–33%), indicating domain divergence within AiCTL-9. However, each of these domains forms a high-identity cluster with the corresponding d1, d2, d3, and d4 domains of the lectins Cnlec-1 from *M. crassicostata* and Cflec-3 from *S. farreri*. For example, AiCTL-9[d1] and Cnlec-1[d1] share 88.46% identity, while AiCTL-9[d4] and Cnlec-1[d4] share 75.54%. This indicates conservation of not just proteins but specific domain architectures across species. A similar pattern is observed for the lectins ScCTL-1 from *S. constricta* and Cflec-4 from *S. farreri*, where the corresponding domains (d1–d4) exhibit high pairwise identity (e.g., ScCTL-1[d4] and Cflec-4[d4] share 42.96% identity, which is high for lectins from different species). PIM also clearly demonstrates the formation of internal evolutionary clusters, among which, in addition to the lectin domains AiCTL-9, Cnlec-1, Cflec-3, ScCTL-1, and Cflec-4 with very high homology, domains with moderately high (25–45%) and low (15–25%) identity can be identified. The group with moderately high homology includes domains such as CgCLec-1 from *M. gigas*, CvML from *C. virginica*, AiCTL-7 from *A. irradians*, AiCTL1 from *A. irradians*, GYL from *G. yessoensis*, RpCTL from *R. philippinarum*, TgCTL-1 from *T. granosa*, Codakine lectin from *Codakia orbicularis*, and CgCLec-2 from *M. gigas*. Of particular note are the lectins CgCLec-1 and CvML, which exhibit very high identity (80.15%), indicating a close evolutionary relationship. Among the domains examined, there are unique sequences, such as CgCLec-5 from *M. gigas* and CgCLec-3 from *M. gigas*, which exhibit exceptionally low identity to all other domains examined (on average, ~2–25%), emphasizing their uniqueness in the studied set. Thus, PIM analysis confirms that the C-type lectin family in bivalves is extremely diversified. Moreover, evolution occurred not only at the level of entire proteins but also at the level of individual domains, with the formation of conserved domain architectures in unrelated species. Multiple alignment of CRD regions of CTLs from bivalves and PIM calculation were performed using Clustal Omega using default protein sequence alignment parameters [106].

**Figure 4:**
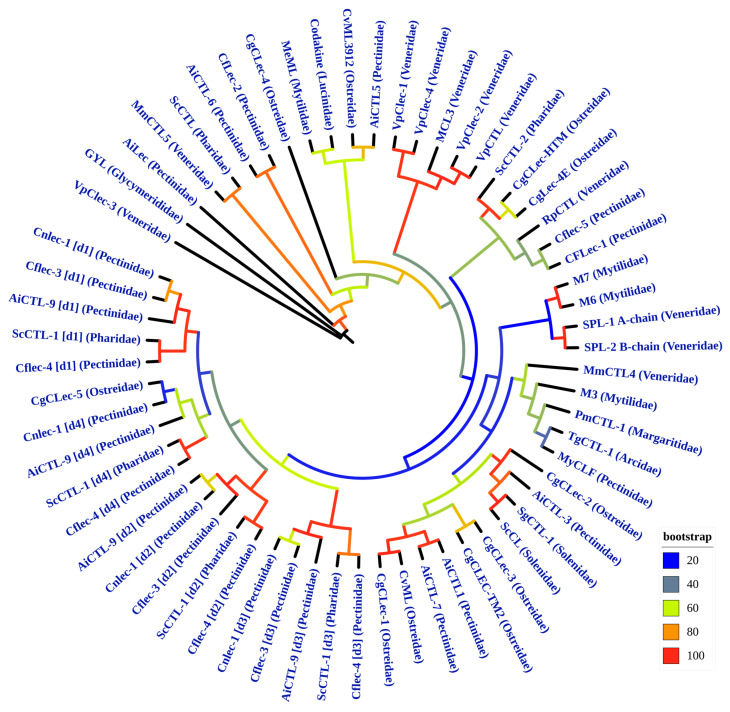
Phylogenetic tree of the CRD regions of CTLs of bivalves.

The phylogenetic tree, constructed with high statistical support, generally confirms and visualizes the patterns revealed by the PIM analysis (Figure 4). The phylogenetic tree clearly identifies large, well-supported monophyletic clades (bootstrap > 90) that unite CRDs from multidomain CTLs. Specific clustering patterns are observed between the d1, d2, d3, and d4 domains of the AiCTL-9, Cnlec-1, Cflec-3, ScCTL-1, and Cflec-4 proteins. The formation of such distinct clades further supports modular evolution and independent domain divergence following duplication events. Furthermore, these domain-specific clades contain domain sequences from different molluscan genera. Importantly, this includes the ScCTL-1 protein from *S. constricta* (family Pharidae), which consistently clusters with domains from proteins of the family Pectinidae (e.g., AiCTL-9, Cflec-4). This indicates that the gene duplications leading to these multidomain lectins occurred before the divergence of the Pectinoidea and Pharidae lineages, and subsequent domain evolution was conservative. Clades of single-domain lectins, such as CgCLec-2, Codakine lectin, RpCTL, and TgCTL-1, form their own clades, often with high support. This suggests that they diverged from common ancestors distinct from the ancestors of domains in multidomain proteins. The unique sequences of CgCLec-3 and CgCLec-5 occupy a basal position and form a long branch in one of the clades, indicating a long phylogenetic distance, consistent with their low identity in the PIM and proposed structural and functional features. Interestingly, despite low amino acid sequence identity, human MBL lectin shares a closer phylogenetic relationship with bivalve CTLs, such as CgCLec-4 and MmCTL5. This indicates deep evolutionary conservation of some CRD domain sequences, descending from a common ancestor. Clustering on the tree directly correlates with high percent identity values in PIM. Domains grouped into a single clade on the tree exhibit high pairwise percent identity. Disparate domains with low identity (e.g., CgCLec-3, CgCLec-5) occupy corresponding isolated positions on the phylogeny. The phylogenetic tree of CTLs reflects, though is not a perfect mirror of, the evolutionary history of the host organisms themselves. While a high degree of gene conservation and co-evolution with bivalve lineages is evident, the observed patterns are also shaped by lineage-specific gene duplications and divergent evolution. A notable pattern is the clustering of orthologous proteins from closely related species. For example, the oyster proteins CvML (*C. virginica*, Ostreidae) and CgCLec-1 (*M. gigas*, Ostreidae) form a well-supported sister pair, indicating common ancestry and divergence after speciation. Similarly, the mussel proteins M6 and M7 (*M. edulis*, Mytilidae) are nearly identical, suggesting a very recent gene duplication. However, the broader grouping including M3 (*M. edulis*, Mytilidae) has low statistical support (bootstrap < 20), indicating an unresolved relationship. In contrast, the lectins from the family Veneridae (subclass Heterodonta) exhibit a complex, non-monophyletic distribution, underscoring multiple independent evolutionary origins or events within this taxon. The proteins VpClec-1, -2, -4, VpCTL, and MCL3 from *R. philippinarum* form a well-supported sub-clade, indicative of recent lineage-specific gene duplications and expansion of a paralogous gene family. However, VpClec-3 from the same species occupies a distinct, separate phylogenetic position on a long branch, suggesting an earlier divergence and possibly a different functional role. The SPL-1 and SPL-2 proteins from *Saxidomus purpurata* (=*Saxidomus purpuratus*) form their own robust pair but are phylogenetically distant from the *R. philippinarum* lectin clusters. Furthermore, the two lectins from *M. meretrix*, MmCTL4 and MmCTL5, are not sister proteins and are placed in two different major clades of the tree. Lastly, RpCTL from *R. philippinarum* clusters separately from all other Veneridae proteins, grouping instead with lectins from the family Pectinidae. This intricate pattern, where lectins from a single molluscan family are scattered across the phylogenetic tree, points strongly to repeated gene duplication events followed by divergent evolution and possible deep paralogy, rather than a simple vertical inheritance followed by speciation-driven divergence. It highlights how the evolution of immune-related genes like CTLs can be decoupled from the phylogeny of the host organisms at the family level [27]. The position of several proteins aligns with broader taxonomic groups. The CTL GYL from *G. yessoensis* (family Glycymerididae, order Arcida) occupies a basal position on a long, separate branch, consistent with the phylogenetic position of Arcida as an early-diverging lineage within the infraclass Pteriomorphia. In contrast, the TgCTL-1 protein from *T. granosa* (Arcidae, also Arcida) clusters within the large Pteriomorphia clade, indicating a different evolutionary trajectory for this lectin within the same order. The Codakine protein from *C. orbicularis* (Lucinidae, order Lucinida) is phylogenetically placed near clusters containing proteins from Veneridae (order Venerida) and Mytilidae (Pteriomorphia). This intermediate placement is consistent with molecular phylogenies that position the Lucinida lineage as an early-diverging group within the subclass Heteroconchia, distinct from but related to the lineage leading to Venerida. Overall, the evolution of CTL genes in bivalves is complex and linked to both the macroevolution of the group and gene-level events. The presence of closely related pairs or groups of nearly identical proteins within a single species (e.g., VpClec-1 and VpClec-4, M6 and M7) indicates recent gene duplications. The primary drivers of CTL evolutionary diversity appear to be a combination of speciation-related divergence of orthologs and lineage-specific expansion and divergence of paralogous families. Thus, while the CTL phylogeny provides valuable insights and confirms certain evolutionary relationships, it also reveals a history influenced by gene duplication and functional innovation, explaining why it does not always strictly correspond to the organismal phylogeny. In conclusion, the phylogenetic tree serves as a tool for studying immune system evolution and as a molecular marker that, when interpreted with caution, can inform our understanding of evolutionary relationships within Bivalvia. The phylogenetic tree was generated using the IQ-TREE software (2.4.0) by the maximum likelihood method with the VT+R4 evolutionary model. Tree topology reliability was assessed using UltraFast Bootstrap (1000 iterations), and the search for the optimal tree was performed iteratively with a stopping criterion of 100 unsuccessful improvement attempts (Nearest Neighbor Interchange). The tree is presented unrooted [107]. All taxonomic classifications mentioned in this study have been verified and standardized according to the authoritative database MolluscaBase. The phylogenetic tree was constructed and visualized in iTOL [108].

Analysis of the motifs responsible for carbohydrate binding and calcium ion coordination reveals the molecular basis for the functional diversity of this class of lectins in bivalves (Table 4). Key residues in CTLs that mediate their carbohydrate-recognition properties are conserved amino acid residues located in the Ca^2+^-binding site 2, which determine carbohydrate specificity and coordinate the geometry of the Ca^2+^-binding site. Variability in the carbohydrate-binding motif (EPN/QPD) determines the binding preferences of CTLs for specific monosaccharide residues in carbohydrates. Thus, the common EPN motif associated with mannose/glucose binding is widely represented in the lectins under study (GYL, MyCLF, VpClec-3, RpCTL, AiCTL-3, Codakine lectin). Another classical motif differentiating the carbohydrate-binding properties of CTLs is the QPD motif, which is associated with galactose binding in CTLs from bivalves (VpClec-1, AiCTL1, M6, and M7). Unique variations in the carbohydrate-binding motif (EPN/QPD) have also been detected, highlighting the plasticity of CTLs from bivalves. Lectin CgCLec-5 has a non-canonical QYE motif, which likely explains its unique position in the PIM and its phylogenetic distance in the tree. Another lectin, CgCLec-3, has a unique DIN motif and lacks a second conserved motif in this binding site that coordinates Ca^2+^. Despite this, the protein is calcium-dependent, suggesting an alternative binding mechanism. SPL-1/SPL-2 lectins have unique RPD/KPD-charged residues (Arg/Lys), which may explain a unique carbohydrate-binding mechanism that is not strictly Ca^2+^-dependent. Furthermore, the CRDs of bivalve mollusks exhibit variability in the calcium-coordinating motif. Most motifs contain a conserved tryptophan, which is critical for stabilizing protein-carbohydrate interactions. The canonical WND, WSD, and WHD motifs are widely represented in the CRDs of bivalve CTLs. However, unique variations in these motifs can be found among the lectins under consideration. For example, this motif is completely absent from the CgCLec-3 lectin, which, however, does not prevent it from being calcium-dependent. This suggests the existence of atypical Ca^2+^ coordination sites. The lectin ScCTL-2 has a WHD motif, but its calcium independence has been experimentally demonstrated. This indicates that the presence of a conserved motif does not always guarantee functional dependence on calcium, and that the overall structure of the domain and variable loops plays a key role. The aforementioned SPL-1/SPL-2 lectins have a canonical WND coordinating motif, but calcium is not strictly required. This is due to a fundamentally new mechanism of calcium-independent carbohydrate recognition through different regulation of the carbohydrate-binding site geometry. The lectin VpClec-2 has an ISG motif, indicating a complete loss of tryptophan, which likely also modulates protein-carbohydrate interactions. In multidomain proteins (AiCTL-9, ScCTL-1, Cflec-4, Cnlec-1), different domains carry different combinations of motifs. For example, in AiCTL-9, CRD1 has YPT/FQN, CRD2 has EPD/FSD, CRD3 has EPN/YND, and CRD4 has QPN/YMV. This allows a single protein to recognize a wide range of different carbohydrate ligands. Motif variability is the primary mechanism underlying the functional diversity of mollusk CTLs. The presence of unique and non-canonical motifs is directly related to the domains’ specialization in binding specific carbohydrate structures, often under conditions different from classical calcium dependence.

**Figure 5:**
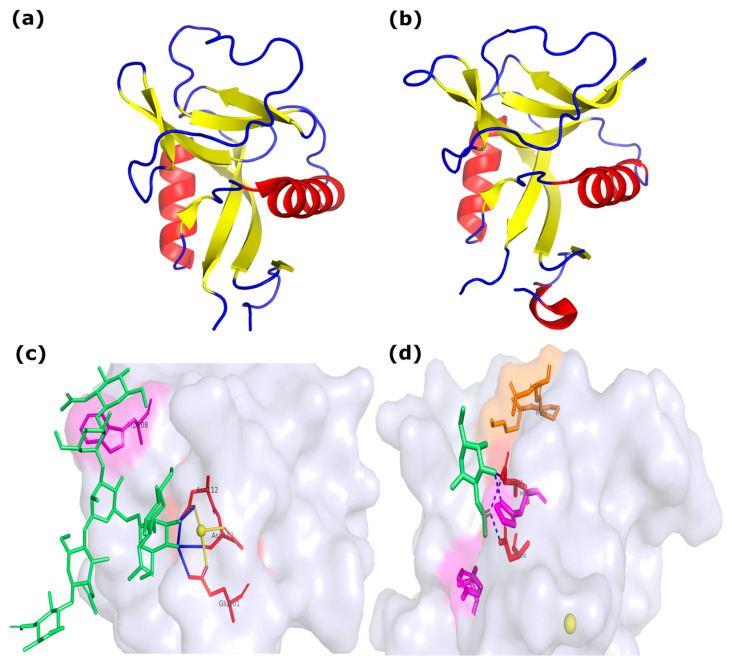
Overview of the crystal structures of CTLs from the bivalves *C. orbicularis* and *S. purpurata*; (**a**) Structural monomer fold of Codakine lectin (PDB 2VUZ): red—α-helix, yellow—β-strand, blue—loop; (**b**) Structural monomer fold of SPL-2 lectin (PDB 6A7S): red—α-helix, yellow—β-strand, blue—loop. (**c**) Carbohydrate binding site in Codakine lectin: green stick—N-glycan (ligand), red sticks—Asn112, Asp113 and Glu101, purple stick—Trp108 (stacking zone), blue lines—hydrogen bonds, yellow sphere and lines—calcium atom and coordinating bonds; (**d**) Carbohydrate binding site in SPL-2 lectin: green stick—GalNAc (ligand), red sticks—Asp106, Asp118, purple sticks—Tyr66 and His120 (stacking zone), blue lines—hydrogen bonds, yellow sphere—calcium atom, brown sticks—“silent” KPD (Lys97-Pro98-Asp99) motif.

To date, the three-dimensional structures of CTLs have been experimentally determined for only two species of marine bivalves, serving as an important reference for understanding the evolution of this protein family. The CTL Codakine, the first structurally characterized CTL from a bivalve, is a homodimer stabilized by a unique covalent interchain disulfide bridge between Cys44 residues. Each monomer contains the canonical β-sandwich of the C, stabilized by three intrachain disulfide bridges, two of which (Cys30–Cys124 and Cys103–Cys116) are conserved within the family (Figure 5a). A key feature of Codakine is the presence of only one of the two conserved calcium ions, at site 2 (the canonical carbohydrate-binding site). Site 1 is absent; its position is structurally occupied by the side chain of Lys70. Carbohydrate binding in Codakine is a classical Ca^2+^-dependent process. It is mediated by the conserved EPN motif (Glu101-Pro102-Asn112), which determines mannose specificity, and the WND motif (Trp105-Asn112-Asp113). The Asn112 and Asp113 residues of the WND motif act as direct ligands for the Ca^2+^ ion at site 2, forming a coordination sphere to which the O3 and O4 hydroxyls of the α1-6-mannose of the biantennary N-glycan bind via calcium. Codakine’s uniqueness lies in the presence of an expanded binding site: the terminal GlcNAc of the α1-3 antenna of the same N-glycan engages in hydrophobic stacking with Trp108 (Figure 5c). This, together with an additional hydrogen bond network, explains the record-breaking submicromolar affinity (Kd ~0.43 nM) for complex glycans, atypical for most CTLs. Thus, Codakine demonstrates evolution toward the refinement and expansion of a classical mechanism on a conserved structural framework. CTLs SPL-1/2 are dimers formed by either two different (SPL-1: a heterodimer of A- and B-chains) or two identical subunits (SPL-2: a homodimer of B-chains) (Figure 5b). Dimerization is also stabilized by interchain disulfide bridges (Cys(A2)-Cys(B47) and Cys(A4)-Cys(B1) in SPL-1). The CRD domain retains the general β-sandwich of CTLDs, stabilized by a set of four intrachain disulfide bonds, including a unique C-terminal bond (Cys1-Cys135 in the A-chain) not found in most CTLs. Functionally, SPL-1 and SPL-2 represent a radical evolutionary departure from the canonical mechanism. Despite the presence of a Ca^2+^ ion in the structure (one per subunit), it is located away from the carbohydrate-binding site and presumably plays a structural role. A key difference is the complete absence of the functional canonical EPN and WND motifs. Instead, the corresponding positions are occupied by the RPD (A-chain) and KPD (B-chain) sequences, which are not involved in ligand binding. The crystal structure of the SPL-2 complex with GalNAc reveals a fundamentally different, Ca^2+^-independent recognition mechanism (Figure 5d). Specificity for N-acetylated sugars (GlcNAc/GalNAc) is ensured by interaction with their acetamide group. It is recognized through stacking interactions with the aromatic residues Tyr66 and His120, as well as through hydrogen bonds with Asp106 and Asn118. The hydroxyl groups of the sugar play a minimal role. Although binding occurs in the absence of Ca^2+^, its presence in solution allosterically enhances the affinity of lectins, likely modulating protein conformation. A comparative analysis of experimental structures of bivalve CTLs clearly illustrates the central evolutionary paradox of this superfamily: extremely high conservatism of the tertiary structure (β-sandwich CRD) is combined with exceptional plasticity of functional mechanisms. Codakine and SPL-2 represent two fundamentally different mechanisms of carbohydrate ligand binding, implemented on a conserved structural framework. Codakine lectin exhibits an evolutionary refinement of the canonical calcium-dependent recognition mechanism, while SPL-2 undergoes a complete functional reconfiguration with the formation of a new calcium-independent binding site. These data provide a compelling experimental justification for studies aimed at identifying similar structural conservatism with variable specificity among a broader set of bivalve lectins.

**Figure 6:**
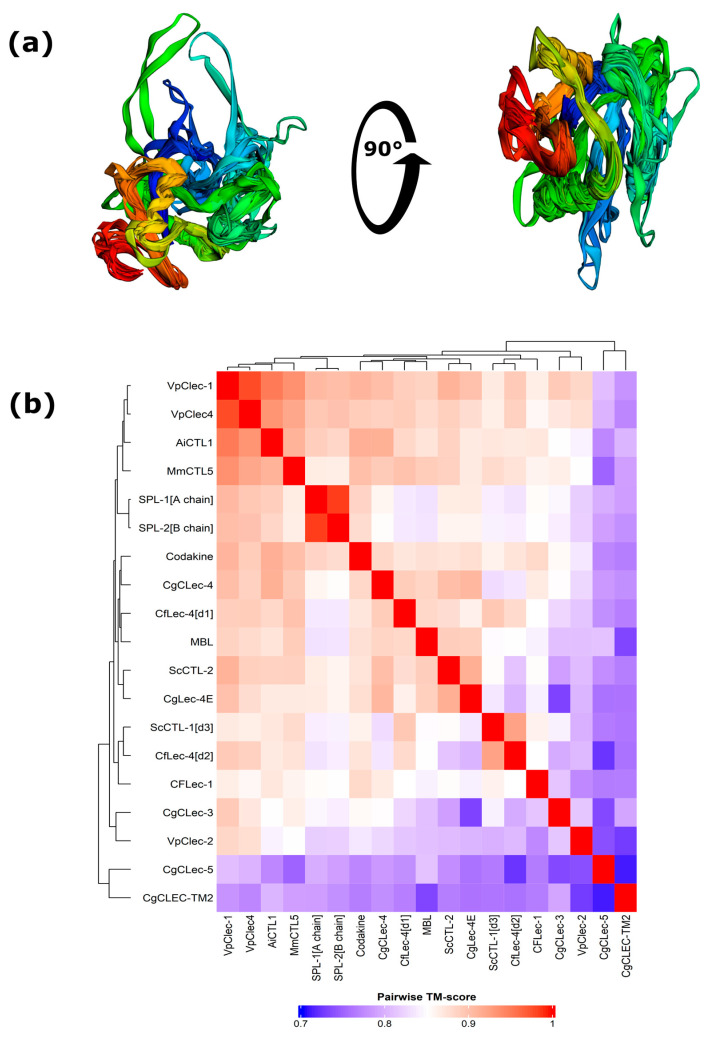
Results of multiple structural alignment of CRD domain models of CTLs from bivalves. (**a**) Structural superposition of CRD domains of CTLs from bivalves; (**b**) pairwise comparison matrix of TM-score for structurally aligned CRD domain structures of CTLs from bivalves.

The availability of crystal structures of invertebrate and vertebrate CTLs enables highly accurate and relevant modeling of the CRD domains of bivalve CTLs using modern diffusion modeling techniques (e.g., AlphaFold, RosseTTAFold). To directly test and confirm the concept of similarity between carbohydrate-recognizing structural scaffolds at low amino acid sequence homology, we first generated high-confidence structural models of CRD domains of CTLs, selected based on their structural and functional diversity, followed by multiple structural alignments to obtain a topological similarity coefficient for CRD structures of CTLs (Figure 6a). Subsequent systematic comparison of these predicted models via multiple structural alignments and TM-score calculation revealed that the overwhelming majority of pairwise comparisons demonstrate a TM score > 0.8 (Figure 6b). This structural metric unequivocally confirms that, despite the low amino acid sequence homology and variability of key functional motifs, the CRD domains of mollusk CTLs retain a common canonical β-sandwich fold at the tertiary structure level.

The heatmap reveals clusters of structurally related domains that generally correspond to phylogenetic clades and groups with high PIM identity. For example, the CRD domains of VpClec-1, VpClec-4, AiCTL1, MmCTL5, and Codakine form a tight cluster with a TM score > 0.9 among themselves. The CRDs of the multidomain proteins ScCTL-1[d3], Cflec-4[d1], and Cflec-4[d2] also exhibit high structural similarity to each other. The CRD structure of CgCLec-3 has relatively low TM scores (~0.73–0.85) with other domains, which structurally confirms its uniqueness revealed at the sequence and motif levels. ScCTL-2 exhibits high structural similarity (TM score ~0.86–0.91) to typical CRDs (MBL, Codakine) despite its calcium independence. This suggests that its unique property is due not to a global fold change, but to specific changes in the binding site. The SPL-1/SPL-2 lectin CRDs have very high structural similarity to each other (TM score 0.984) and to other lectins, consistent with conservation of the overall fold even if their binding is not strictly calcium-dependent. Interestingly, clustering of Codakine lectin and SPL-1/2 lectins with fundamentally different modes of carbohydrate recognition into different hierarchical groups by TM score may have predictive power, which opens the possibility for targeted study of the pool of molecular mechanisms of carbohydrate ligand recognition. Structural analysis supports the concept of conserved carbohydrate-recognition folds with flexible functional sites. Evolution has created a wide diversity of functions, primarily modifying key motifs in the binding sites, while the overall domain architecture has remained stable. Thus, the evolution of CTLs in bivalves occurred largely at the domain level. Multidomain proteins arose through ancient duplications, and their individual domains evolved independently, forming conserved, domain-specific evolutionary lineages that have been preserved in unrelated species.

Our integrated analysis combining sequence-based phylogeny, motif identification, and, crucially, high-accuracy structural modeling reveals a clear evolutionary picture. While sequence divergence is high, the core β-sandwich fold, as revealed by comparative analysis of AlphaFold2-predicted structures, remains strikingly conserved (TM-score ~0.73–0.984). The primary source of functional diversity is therefore the variability of key motifs responsible for carbohydrate specificity (EPN/QPD and their unique variants) and calcium coordination (WND and its unique variants/WSH and others) embedded within this stable structural framework. Furthermore, we observe that calcium-dependent carbohydrate recognition is no longer the sole mechanism describing the carbohydrate specificity of these lectins. These data paint a portrait of a protein family with an exceptionally flexible and adaptive evolutionary strategy, which allows bivalves to effectively utilize CTLs to perform a wide range of physiological functions.

To enable a structural comparison and assess fold conservation independent of sequence similarity, the 3D structures of the CRD domains were predicted using ColabFold v1.5.2 (compatible with AlphaFold2). MMseqs2 was used with the UniRef and Environmental databases to search for homologous sequences. The model was built in «unpaired_paired» mode with a greedy MSA assembly strategy. The AlphaFold2_ptm model with three rounds of refinement (recycling) was used. Up to 256 sequences were selected from the multiple alignment for the single MSA and 512 for the paired MSA. The predicted structures were not relaxed. Models with the highest confidence scores (pLDDT, pTM) were selected for subsequent structural alignment and TM-score analysisCTLs [113]. Multiple structural alignment and TM-score calculation were performed on the mTM-align server [114].

## 6. Carbohydrate Specificity of C-Type Lectins

The carbohydrate-binding specificity of CTLs is determined by the presence of a carbohydrate-recognition domain (CRD), which is often, but not always, dependent on calcium ions (Ca^2+^). It was traditionally believed that binding specificity is predetermined by key amino acid motifs in the Ca^2+^-binding site 2: the EPN motif is associated with the recognition of mannose, glucose, or N-acetylglucosamine, while the QPD motif is associated with the recognition of galactose and its derivatives. However, as studies on CTLs in mollusks show, this classification is simplified, and their carbohydrate specificity is much more complex and diverse [35,47,61,112,115]. Recent studies have identified numerous CTLs in bivalve mollusks with unique binding profiles that do not always fit predictions based on the primary structure of their CRD (Table 4).

The lectin GYL from the mollusk *G. yessoensis* demonstrates high and selective affinity for only a limited range of carbohydrates and glycoproteins. The strongest binding is observed with the tetrasaccharide GalNAcα1-3Galβ1-4(Fucα1-3)GlcNAcβ, and a strong binding reaction was also demonstrated with the epitope 4-O-Su-Galβ1-4GlcNAcβ-sp3, and a weaker reaction with 4,6-O-Su2-Galβ1-4GlcNAcβ-sp2 and GalNAcα1-3(Fucα1-2)Galβ1-4GlcNAcβ-sp3. Analysis of GYL specificity revealed a key disaccharide motif, Galβ1-4GlcNAc, common to all recognized glycans. It was also found that galactose substituents significantly modulate affinity for glycans. For instance, sulfation at the 4-position of galactose enhances binding six-fold, whereas additional sulfation at the 6-position suppresses it. In the case of the most affine tetrasaccharide, fucosylation of the terminal GlcNAc enhanced the binding reaction, while fucosylation of Gal led to a reduction in the reaction. Interestingly, the presence of the “mannose-type” EPN motif in GYL did not facilitate mannose binding, indicating the influence of other structural elements on specificity. Among monosaccharides, GYL exhibited affinity for L-fucose (0.17 mM), and among glycoproteins for the following: porcine submaxillary mucin (PSM, 0.033 mg/mL), asialo-PSM (0.008 mg/mL), fetuin (0.008 mg/mL), asialofetuin (0.004 mg/mL), thyroglobulin (0.004 mg/mL), and ovalbumin (0.025 mg/mL). GYL is likely capable of recognizing O- and N-linked carbohydrate chains and interacting with terminal galactose residues, as treatment of sialoglycoproteins with sialidase significantly increased the lectin’s affinity for desialylated glycoproteins [94,112]. The lectin rMCL3 from *R. philippinarum* demonstrated moderate affinity for raffinose (27 mM) and N-acetylgalactosamine (27 mM), but not for galactose, while the mucin type II (Galβ1,3GalNAcβ1,6GlcNAc-Ser/Thr) completely inhibited bacterial agglutination at a concentration of 0.67 mM [52].

Many mollusks possess lectins with atypical motifs, which expands the repertoire of their carbohydrate specificity. The lectin AiCTL-7 from the scallop *A. irradians* contains an EPN motif where asparagine is replaced by aspartic acid. Despite this substitution, the recombinant protein (rAiCTL-7) retained the ability to bind mannose, as confirmed by the inhibition of *P. pastoris* yeast agglutination by D-mannose (200 mM), but not by galactose. However, in the case of AiCTL-7 with the EPD motif, where asparagine was replaced by aspartic acid, rAiCTL-7 could bind both D-mannose (200 mM) and D-galactose (200 mM). Thus, the substitution of asparagine with aspartic acid does not change the specificity from mannose to galactose [35,93]. In another study, a CTL from the Pacific oyster *M. gigas*, CgCLec-3, was found to contain a novel DIN motif, previously unreported in invertebrate CTLs, and possesses moderate affinity for mannose (0.45 mM) [41].

The lectin MmCTL5 from *M. meretrix* has QPS and WND motifs. It demonstrates broad but weak specificity, binding glucose, D-mannose, D-xylose, D-galactose, lactose, and maltose with high minimum inhibitory concentration values (100–200 mM) [47]. Another lectin, MmCTL4 from *M. meretrix*, contained QPN and WSD motifs in its Ca^2+^-binding sites and bound D-mannose, D-xylose, lactose, and maltose with minimum inhibitory concentrations of 100 mM, 25 mM, 100 mM, and 100 mM, respectively, but did not bind D-galactose or glucose [46]. The multidomain lectin Cflec-3 from *S. farreri* contains three CRDs with different motifs: CRD1 (YPT), CRD2 (EPD), and CRD3 (EPN). Despite this, the entire protein overall exhibits mannose specificity. This proves that the EPD motif in CRD2 can support mannose binding (200 mM), and the specificity of the whole protein is determined by the combination of all its domains [61].

Some bivalve lectins follow classical predictions, while others demonstrate complete deviation from them. Codakine from *C. orbicularis* with an EPN motif confirmed its specificity for D-mannose (25 mM) and L-fucose (25 mM) and also exhibited weaker affinity for glucose (100 mM) and N-acetylglucosamine (100 mM). In the case of oligosaccharides, only 13 carbohydrates had a high degree of affinity and represent complex-type biantennary N-glycans [115]. Unlike codakine, AiCTL-3 from *A. irradians*, which also has an EPN motif, is capable of binding both D-mannose (0.035 mM) and D-galactose (0.035 mM) [33].

CTLs have been found that lack dependence on Ca^2+^ ions. The lectins SPL-1/SPL-2 from *S. purpurata* were Ca^2+^-independent and exhibited affinity for GlcNAc (5 mM) to a greater extent and for GalNAc (25 mM) to a lesser extent, but not for glucose or galactose. Oligosaccharides containing GlcNAc or GalNAc with α-glycosidic linkages possess fairly high affinity for both CTLs [110]. Similarly, CfLec-2 from *S. farreri* agglutinated bacteria independently of Ca^2+^, and its activity was inhibited only by D-mannose (200 mM) [98]. Another Ca^2+^-independent CTL from *S. farreri*, CfLec-5, also exhibited high affinity for D-mannose [65].

The carbohydrate specificity of CTLs in mollusks represents a combination of patterns: from strict specificity for individual monosaccharides to high-affinity recognition of complex, modified oligosaccharide epitopes. This diversity appears to be a result of the adaptation of the mollusk’s immune system, which allows it to effectively recognize a wide range of pathogenic microorganisms in the aquatic environment.

Recently, carbohydrate-binding proteins from marine organisms have been considered promising molecular tools for detecting aberrant glycosylation patterns in tumor cells [116]. A significant gap in the study of the functional properties of the examined CTLs from bivalve mollusks is the lack of detailed information on their carbohydrate specificity for clinically relevant glycans. Our systematic analysis revealed that CTLs represent a structurally conserved family of proteins with highly variable carbohydrate-binding motifs, suggesting fine modulation of their carbohydrate specificity. However, we do have extremely interesting information regarding the carbohydrate specificity of some CTLs from bivalve mollusks. Thus, the GYL lectin is capable of binding sulfated forms of lactosamine with high affinity, making GYL a promising scaffold for protein engineering to generate a pool of proteins that differentially recognize sulfated glycans at different positions [112]. This is especially important given emerging information on the role of sulfated glycans in carcinogenesis. Sulfated glycans, including glycosaminoglycans, play a key role in controlling the directed migration of cancer cells [117]. GYL is characterized by combinatorial recognition of complex epitopes based on the oligosaccharide lactosamine, and its carbohydrate specificity is not limited to sulfated glycan derivatives; GYL also recognizes blood group antigens (blood group antigen type A containing GalNAc) and Lewis X antigens, which are involved in cell adhesion and inflammation processes and are often dysregulated in cancer [118,119].

Another C-type lectin from *C. orbicularis* exhibits unique carbohydrate specificity for biantennary complex N-glycans. The interaction of Codakine lectin with biantennary complex N-glycans is exceptionally high-affinity, as has been reported for lectins of this family. A notable feature of these glycans was the absence of core fucosylation of chitobiose in N-glycans. Codakine lectin also recognized the core pentasaccharide Man_3_GlcNAc_2_ and N-glycans elongated by the addition of GlcNAc, galactose, and sialic acid. Biantennary complex N-glycans have recently been considered as biomarkers associated with tumor progression processes [120]. While many lectins recognize terminal sugars (e.g., galactose or sialic acid) on N-glycan arms, Codakine binds with high affinity to the interior of the biantennary structure, specifically to the core pentasaccharide Man_3_GlcNAc_2_, which, among other things, opens the way to the possibility of developing recombinant variants specific for paucimannose N-glycans, which are involved in the metastasis process [121].

Bivalve C-type lectins are of exceptional interest as tools for the detection of tumor-associated glycans due to their unique specificity and serve as valuable scaffolds for protein engineering to create highly specific tools for the detection of aberrant glycosylation in cancer.

## 7. Conclusions

This article provides a detailed review of the structural and functional features of CTLs in bivalves studied to date. For nearly all of them, an immune function and the ability to recognize PAMPs have been demonstrated. In a number of cases, their structure, as well as transcriptional regulation in various conditions and tissues, has been studied with varying degrees of detail. Surprisingly, despite this natural diversity and the multitude of publications, a detailed investigation of their carbohydrate specificity spectrum is extremely rare and is typically limited to one or a few key monosaccharides. However, carbohydrate specificity determines not only the natural functions of lectins but also their potential applications, encompassing the vast field of biomedical glycobiology. In turn, the effective development of methods for modeling the structure and dynamics of biomolecules, as a predictive basis for molecular bioengineering, requires both structural and functionally verified data. Therefore, a shift in the focus of functional studies on bivalve CTLs is required—from merely confirming their immune role towards elucidating the intricacies of their target glycan recognition.

## Figures and Tables

**Table 1:** Constitutive tissue-specific expression profiles of C-type lectins in bivalves.

Species	C-Type Lectin	Hm	Hp	Gn	Mn	Gl	Ad	Others	Indicator	Method	Ref.
*Anadara* *kagoshimensis* (=*Scapharca subcrenata*)	SsCTL1	0	23	1	1	13	70	foot: 0	RE (2^−ΔΔCt^)	RT-qPCR	[31]
	SsCTL2	0	2	0	0	0	0	foot: 0	RE (2^−ΔΔCt^)	RT-qPCR	[31]
	SsCTL3	1	13	9	1	7	2	foot: 9	RE (2^−ΔΔCt^)	RT-qPCR	[31]
	SsCTL4	0	4	5	0	3	40	foot: 65	RE (2^−ΔΔCt^)	RT-qPCR	[31]
	SsCTL5	1	1	2	8	27	1	foot: 4	RE (2^−ΔΔCt^)	RT-qPCR	[31]
*Argopecten* *irradians*	AiCTL1	strong	0	0	0	weak	0	intestine: 0 heart: 0		RT-PCR	[32]
	AiCTL-3	1	1195	10	1	3	4	heart: 1	RE vs. Hm	RT-qPCR	[33]
	AiCTL-6	1	546	48	3	143	8	heart: 33	RE (2^−ΔΔCt^) vs. Hm	RT-qPCR	[34]
	AiCTL-7	21	551	36	N/D	N/D	1	kidney: 44 heart: 11	RE (2^−ΔΔCt^) vs. Hm	RT-qPCR	[35]
	AiCTL-9	1	580	562	64	2	18		RE (2^−ΔΔCt^) vs. Ad	RT-qPCR	[36]
	AiLe	weak	strong	0	0	0	0			RT-PCR	[37]
*Crassostrea* *virginica*	CvML	0	strong	weak	moderate	moderate	0	palp: moderate		RT-PCR, ISH	[38]
*Magallana gigas* (=*Crassostrea gigas*)	CgCLec-1	0	moderate	0	0	0	0	heart: 0 palp: 0		RT-PCR	[39]
	CgCLec-2	3	26	N/D	210	1	2		RE (2^−ΔΔCt^) vs. Gl	RT-qPCR	[40]
	CgCLec-3	N/D	0	moderate	strong	moderate	0			RT-PCR	[41]
	CgCLec-4	27	1433	1	110	2	6		RE (2^−ΔΔCt^) vs. Gn	RT-qPCR	[42]
	CgCLec-5	104	2	1	2	5	10		RE (2^−ΔΔCt^) vs. Gn	RT-qPCR	[42]
	CgCLec-CCP	21	1	12	7	5	1		RE (2^−ΔΔCt^) vs. Hp	RT-qPCR	[30]
	CgCLEC-TM2	94	3	N/D	6	5	1	palp: 4	RE (2^−ΔΔCt^) vs. Ad	RT-qPCR	[43]
	Salivary c-type lectin	0.5	1	N/D	2	9	2	palp: 2	RE (2^−ΔΔCt^)	RT-qPCR	[44]
	C-type lectin 4	1	0.5	N/D	1	5	2	palp: 0	RE (2^−ΔΔCt^)	RT-qPCR	[44]
	CgLec-4E	20	320	15	10	1	1		RE (2^−ΔΔCt^) vs. Gl	RT-qPCR	[45]
	Macrophage mannose receptor 1	1	2	N/D	2	1	0.8	palp: 6	RE (2^−ΔΔCt^)	RT-qPCR	[44]
	C-type mannose receptor 2	1	2	N/D	1	1	0.8	palp: 9	RE (2^−ΔΔCt^)	RT-qPCR	[44]
*Meretrix* *meretrix*	MmCTL4	6	3	N/D	3	2	1	foot: 1	RE (2^−ΔΔCt^) vs. foot	RT-qPCR	[46]
	MmCTL5	4	8	N/D	3	3	1	foot: 2	RE (2^−ΔΔCt^) vs. Ad	RT-qPCR	[47]
*Mimachlamys crassicostata* (=*Chlamys* *nobilis*)	Cnlec-1	120	N/D	55	60	4	1	intestine: 6 kidney: 12	RE (2^−ΔΔCt^) vs. Ad	RT-qPCR	[48]
*Mizuhopecten yessoensis*	MyCLF	0.1	0.04	N/D	1.1	0.4	0.09	kidney: 0.4	RE	RT-qPCR	[49]
*Mytilus edulis*	MeML	0	N/D	0	strong	strong	N/D	intestine: weak palp: strong		ISH	[50]
*Pinctada fucata martensii*	PmCTL-1	1	2	1	33	1	2		RE (2^−ΔΔCt^)	RT-qPCR	[51]
*Ruditapes* *philippinarum* (=*Venerupis philippinarum*)	MCL3	N/D	N/D	N/D	1.3	1	0.4	foot: 1 palp: 0.3 siphon 3.6	Normalized fold expression	RT-qPCR	[52]
	RpCTL	N/D	49	N/D	0.5	4	0.1	foot: 1 siphon: 4	RE (2^−ΔΔCt^) vs. foot	RT-qPCR	[53]
	VpClec-1	62	105,000	N/D	2	80	1		RE (2^−ΔΔCt^) vs. Ad	RT-qPCR	[54]
	VpClec-2	25	80,000	N/D	1	48	1		RE (2^−ΔΔCt^) vs. Ad	RT-qPCR	[54]
	VpClec-3	610	12,000	N/D	1	80	1	foot: 1	RE (2^−ΔΔCt^) vs. muscle	RT-qPCR	[55]
	VpClec-4	280	50	N/D	10	2000	1	foot: 1	RE (2^−ΔΔCt^) vs. muscle	RT-qPCR	[55]
	VpCTL	1.4	1.6	N/D	1	1.5	0.3	foot: 0.8	RE (2^−ΔΔCt^)	RT-qPCR	[56]
	CTL-1	N/D	9	6	5	12	0.6	foot: 1 siphon: 4	RE (2^−ΔΔCt^) vs. foot	RT-qPCR	[57]
	CTL-2	N/D	115	21	4	51	5	foot: 1 siphon: 28	RE (2^−ΔΔCt^) vs. foot	RT-qPCR	[57]
	CTL-3	N/D	0.078	0.083	0.005	0.76	0.001	foot: 1 siphon: 0.007	RE (2^−ΔΔCt^) vs. foot	RT-qPCR	[57]
	CTL-4	N/D	36	11	0.6	6	0.4	foot: 1 siphon: 0.6	RE (2^−ΔΔCt^) vs. foot	RT-qPCR	[57]
	CTL-5	N/D	18	4	15	50	0.5	foot: 1 siphon: 5	RE (2^−ΔΔCt^) vs. foot	RT-qPCR	[57]
	CTL-6	N/D	106	9	10	79	0.9	foot: 1 siphon: 2	RE (2^−ΔΔCt^) vs. foot	RT-qPCR	[57]
*Scaeochlamys farreri* (=*Chlamys* *farreri*)	CFLec-1	weak	0	moderate	weak	strong	0	kidney: 0.4		RT-PCR	[58]
		moderate	0	0	moderate	moderate	0	kidney: 0 foot: 0		IHC	[59]
	CfLec-2	moderate	0	moderate	moderate	0	0	kidney: moderate foot: 0		IHC	[60]
	Cflec-3	1	555	32	400	37	419		RE (2^−ΔΔCt^)	RT-qPCR	[61]
		moderate	moderate	N/D	moderate	moderate	moderate	kidney: moderate foot: moderate		IHC	[62]
	CfLec-4	N/D	moderate	moderate	N/D	0	0			IHC	[63]
		0	71	1	0	0	0		RE (2^−ΔΔCt^)	RT-qPCR	[64]
	Cflec-5	1	104	358	695	943	17		RE (2^−ΔΔCt^) vs. Hm	RT-qPCR	[65]
*Sinonovacula constricta*	ScCL	0.1	0.8	N/D	1.7	1.9	N/D	intestine: 0.7 foot: 3.4 palp: 2.1 siphon: 1.4	RE (2^−ΔΔCt^)	RT-qPCR	[66]
	ScCTL	117	708	N/D	N/D	360	N/D	foot: 1 siphon: 47	RE vs. foot	RT-qPCR	[67]
	ScCTL-1	8	19,939	42	2	11	N/D	foot: 5 siphon: 1	RE (2^−ΔΔCt^) vs. siphon	RT-qPCR	[68]
	ScCTL-2	3	1	N/D	N/D	4	N/D	foot: 109 siphon: 27	RE (2^−ΔΔCt^)	RT-qPCR	[69]
*Solen grandis*	SgCTL-1	1	59	17	1	86	3		RE (2^−ΔΔCt^) vs. Mn	RT-qPCR	[70]
*Tegillarca granosa*	TgCTL-1	360	4000	N/D	5	200	1	foot: 1	RE (2^−ΔΔCt^) vs. muscle	RT-qPCR	[71]

Hm—hemocytes; Hp—hepatopancreas; Ad—adductor; Mn—mantle; Gn—gonad; Gl—gill; RE vs.—relative expression compares to; RT-qPCR—reverse transcription quantitative polymerase chain reaction; N/D—not determined.

**Table 2:** RT-qPCR analysis of C-type lectins expression dynamics in bivalves following immunostimulation.

Species	C-Type Lectin	Stimulation	Tissue	0 h	3 h	6 h	12 h	24 h	48 h	Ref.
*A. kagoshimensis*	SsCTL1	*Vibrio* *parahaemolyticus*	Hp	1.4	5.8	505	282	134	59.5	[31]
	SsCTL2	*V. parahaemolyticus*	Hp	1.1	2.1	149	119	149	64.9	[31]
	SsCTL3	*V. parahaemolyticus*	Hp	1	2.6	0.6	0.5	2.4	0.1	[31]
	SsCTL4	*V. parahaemolyticus*	Hp	1.1	9.6	65.6	133.3	3.4	65.6	[31]
	SsCTL5	*V. parahaemolyticus*	Hp	1.4	−9.5	−2118	−1177	−179	−2588	[31]
*A. irradians*	AiCTL-3	*V. anguillarum*	Hm *	1	1.5	1.5	N/D	N/D	1.5	[33]
		*M. luteus*	Hm *	1	2	3	N/D	N/D	1	[33]
	AiCTL-6	*M. luteus*	Hm *	1	1.4	31.9	33.1	2.4	1.1	[34]
		*L. anguillarum*	Hm *	1	3.8	6.4	1	1	1	[34]
	AiCTL-7	*P. pastoris*	Hm	1	9	19	95	2	N/D	[35]
			Hp	1	4	16	1	2	N/D	[35]
		*L. anguillarum*	Hm	1	3	9	12	1	N/D	[35]
			Hp	1	4	8	30	3	N/D	[35]
	AiCTL-9	*V. anguillarum*	Hm	1	0.5	0.3	6.2	1.2	N/D	[36]
		*P. pastoris*	Hm	1	0.7	3.2	2.6	1.7	N/D	[36]
		*M. luteus*	Hm	1	1.9	1.4	0.8	2.2	N/D	[36]
*M. gigas*	CgCLec-2	*Vibrio splendidus*	Hm *	1	1.1	4	3	1.2	N/D	[40]
	CgCLec-3	*V. splendidus*	Hm *	1	1.2	6.7	3.6	1.2	N/D	[41]
		*V. anguillarum*	Hm *	1	1.2	6.9	2.7	1.2	N/D	[41]
		LPS	Hm *	1	1.3	3.1	1.3	1.3	N/D	[41]
		PGN	Hm *	1	1.2	6.2	1.6	1	N/D	[41]
		β-glucan	Hm *	1	1.3	14	1.6	1	N/D	[41]
	CgCLec-4	LPS	Hm *	1	1.5	3.1	0.8	1	N/D	[42]
	CgCLec-5	LPS	Hm *	1	0.9	1.7	2.8	1.3	N/D	[42]
	CgCLec-CCP	*V. splendidus*	Hm *	1	2	4.8	16	7.8	7.8	[30]
	CgCLEC-TM2	*V. splendidus*	Hm *	0.9	N/D	7.6	0.4	8	1.2	[43]
	CgLec-4E	*Vibrio alginolyticus*	Hp *	1	N/D	1.7	1.2	0.7	1.1	[45]
*M. meretrix*	MmCTL4	*V. splendidus*	Hm	1	1.2	9.2	1.8	1.7	1	[46]
			Hp	1	0.6	1.4	4.9	0.9	0.9	[46]
	MmCTL5	*V. splendidus*	Hm	1	1.2	1.1	6.4	2.4	1	[47]
			Hp	1	9	7.5	3.2	1	1.3	[47]
*M. yessoensis*	MyCLF	*V. anguillarum*	Hm *	0.8	5.6	0.4	0.6	0.1	N/D	[49]
	MCL3	*Vibrio tapetis*	Hm *	N/D	N/D	N/D	N/D	0.5	2	[52]
		*P. olseni*	Hm *	N/D	N/D	N/D	N/D	0.2	0.3	[52]
*R. philippinarum*	VpClec-1	*V. anguillarum*	Hm	0.8	N/D	2.3	0.3	80	1.7	[54]
	VpClec-2	*V. anguillarum*	Hm	0.9	N/D	5	16	24	1.2	[54]
	VpClec-3	*V. anguillarum*	Hm	1	N/D	1.9	0.2	6.1	4.3	[55]
			Hp	1	N/D	777	693	687	2.4	[55]
			Gill	1	N/D	10.2	4.5	0.9	1.1	[55]
	VpClec-4	*V. anguillarum*	Hm	1	N/D	4.7	0.1	1.4	1.4	[55]
			Hp	1	N/D	1.5	179	1.8	1.3	[55]
			Gill	1	N/D	23.6	25.9	12.5	3.2	[55]
	VpCTL	*L. anguillarum*	Hm *	1	N/D	1.7	3.3	3.6	107	[56]
*S. farreri*	CfLec-1	LPS	Hm	1	2	2.9	5.7	4.5	3.4	[59]
		β-glucan	Hm	1	3.3	2.7	4.3	6	2.6	[59]
		PGN	Hm	1	0.1	0.2	1.1	2.2	1	[59]
	CfLec-2	LPS	Hm	N/D	0	0	70	5	2	[60]
		PGN	Hm	N/D	0	3	24	10	5	[60]
		β-glucan	Hm	N/D	24	17	43	30	4	[60]
	CfLec-3	LPS	Hm	1	0.9	13	11	3	0	[62]
		PGN	Hm	1	3	8	25	12	2	[62]
		β-glucan	Hm	1	28	7	47	10	4	[62]
		Poly I:C	Hm	1	1	1.8	0.5	0.8	1	[62]
	CfLec-4	PGN	Hm	1	18	3	36	30	1	[63]
		β-glucan	Hm	1	33	3	19	2	3	[63]
		LPS	Hm	1	0.5	22	8	2	0.5	[63]
	Cflec-5	LPS	Hm	1	7	12.5	3.5	4	0.8	[65]
		β-glucan	Hm	1	1	1.5	5.5	1.6	0.6	[65]
		PGN	Hm	1	1	0.3	1.5	1.4	0.4	[65]
		*Staphylococcus* *aureus*	Gl	N/D	2.6	1	10	N/D	23	[66]
	ScCTL	*V. parahaemolyticus*	Hp	1	N/D	N/D	1.3	1.8	1.7	[67]
			Gl	1	N/D	N/D	20.4	3.3	8.8	[67]
	ScCTL-2	*V. parahaemolyticus*	Hp	N/D	1	3.6	3.3	1.6	N/D	[69]
			Gl	N/D	1	2.6	4.4	2.5	N/D	[69]
*S. grandis*	SgCTL-1	LPS	Hm	N/D	1	7.5	10	1	1	[70]
		β-glucan	Hm	N/D	3.5	3.3	22	4.3	0.5	[70]
		PGN	Hm	N/D	50	2200	0	0	0	[70]
*T. granosa*	TgCTL-1	*V. parahaemolyticus*	Hm	1	3.7	12.4	6.9	7.8	3.9	[71]

Hm—hemocytes; Hp—hepatopancreas; Gl—gill; N/D—not determined; *—tissue for which expression data at additional time points are available in the original study.

**Table 3:** Evaluation of C-type lectins’ immunological functions.

Species	C-Type Lectin	Agglutination and Binding Activity	Phagocytic Activity	Hemocytes Encapsulation	Growth Inhibition	PAMPs Binding	Ref.
*A. irradians*	AiCTL-3	*S. aureus* (G+), *V. anguillarum* (G−), *Escherichia coli* (G−)	2.08-fold increasing	N/D	N/D	glucan, LPS, PGN	[33]
	AiCtl5	*E. coli* (G−), *L. anguillarum* (G−)	N/D	N/D	N/D	LPS	[92]
	AiCTL-6	*S. aureus* (G+), *M. luteus* (G+), *E. coli* (G−)	N/D	N/D	N/D	N/D	[34]
	AiCTL-7	*S. aureus* (G+), *V. anguillarum* (G−), *E. coli* (G−), *P. pastoris* (fungi), *Yarrowia lypolytica* (fungi)	N/D	N/D	*E. coli* (G−)	LPS, PGN, β-glucan, poly(I:C), mannan,	[35]
							[93]
	AiCTL-9	*Bacillus subtilis* (G+), *E. coli* (G−), *V. anguillarum* (G−), *P. pastoris* (fungi)	N/D	Increasing up to 97% after 6 h	N/D	LPS, PGN	[36]
*Glycymeris yessoensis*	GYL	*B. subtilis* (G+), *S. aureus* (G+), *E. coli* (G−), *Vibrio proteolyticus* (G−)	N/D	N/D	N/D	LPS, PGN, β-glucan	[94]
*M. gigas*	CgCLec	*S. aureus* (G+), *M. luteus* (G+), *E. coli* (G−), *Sanopus splendidus* (G−)	N/D	N/D	N/D	LPS, PGN,	[30]
	CgCLec-2	*S. aureus* (G+), *V. anguillarum* (G−), *V. splendidus* (G−), *Y. lypolytica* (fungi)	1.66-fold increasing	N/D	*S. aureus* (G+)	LPS, PGN, mannan	[40]
	CgCLec-3	*S. aureus* (G+), *M. luteus* (G+), *V. anguillarum* (G−), *V. splendidus* (G−), *E. coli* (G−), *Y. lypolytica* (fungi), *P. pastoris* (fungi)	3.64-fold increasing	N/D	*S. aureus* (G+), *E. coli* (G−)	LPS, mannan, poly(I:C), lipoteichoic acid	[41]
	CgCLec-4	*S. aureus* (G+), *E. coli* (G−), *V. anguillarum* (G−), *Y. lypolytica* (fungi)	N/D	N/D	*S. aureus* (G+), *E. coli* (G−), *V. algynolyticus* (G−), *Vibrio vulnificus* (G−), *Pseudomonas aeruginosa* (G−), *Y. lypolytica* (fungi)	LPS, PGN, mannan	[42]
	CgLec-4E	*V. alginolyticus* (G−)	N/D	N/D	*V. alginolyticus* (G−)	N/D	[45]
	CgCLec-5	*S. aureus* (G+), *E. coli* (G−), *V. anguillarum* (G−), *Y. lypolytica* (fungi)	N/D	N/D	*E. coli* (G−)	LPS, PGN, mannan	[42]
	CgCLec-HTM	*S. aureus* (G+), *M. luteus* (G+), *E. coli* (G−), *V. anguillarum* (G−)	N/D	N/D	N/D	LPS, PGN	[95]
	CgCLEC-TM2	*B. subtilis* (G+), *S. aureus* (G+), *M. luteus* (G+), *V. anguillarum* (G−), *E. coli* (G−), *V. splendidus* (G−), *P. pastoris* (fungi)	1.3-fold decreasing with anti-CgCLEC-TM2-CRD	N/D	*E. coli* (G−), *V. splendidus* (G−)	LPS, PGN, poly(I:C)	[43]
*M. meretrix*	MmCTL4	*S. aureus* (G+), *B. subtilis* (G+), *E. coli* (G−), *V. parahaemolyticus* (G−), *P. pastoris* (fungi)	N/D	N/D	N/D	LPS	[46]
	MmCTL5	*V. parahaemolyticus* (G−)	N/D	N/D	N/D	N/D	[47]
*P. martensii*	PmCTL-1	N/D	N/D	N/D	*M. luteus* (G+), *S. aureus* (G+), *B. subtilis* (G+)	N/D	[51]
*R. philippinarum*	RpCTL	N/D	N/D	N/D	*S. aureus* (G+), *B. subtilis* (G+), *E. coli* (G−), *V. anguillarum* (G−)	N/D	[53]
	VpClec-1	*V. splendidus* (G−), *Vibrio harveyi* (G−), *V. anguillarum* (G−), *Enterobacter cloacae* (G−), *Aeromonas hydrophila* (G−)	N/D	Increasing up to 54% after 6 h and up to 90% after 24 h.	N/D	LPS, PGN, glucan, zymosan	[54]
	VpClec-2	*V. splendidus* (G-), *V. harveyi* (G-), *V. anguillarum* (G-), *E. cloacae* (G-), *A. hydrophila* (G-)	N/D	Increasing up to 46% after 6 h and up to 90% after 24 h	N/D	LPS, glucan, zymosan	[54]
	VpClec-3	*S. aureus* (G+), *E. coli* (G−), *V. parahaemolyticus* (G−), *V. harveyi* (G−), *Pseudomonas putida* (G−), *Proteus mirabilis* (G−), *P. pastoris* (fungi)	1.9-fold increasing	N/D	*S. aureus* (G+), *V. splendidus* (G−), *V. harveyi* (G−), *V. anguillarum* (G−), *E. cloacae* (G−), *A. hydrophila* (G−)	PGN	[55]
	VpClec-4	*S. aureus* (G+), *E. coli* (G−), *V. parahaemolyticus* (G−), *V. harveyi* (G−)	1.6-fold increasing	N/D	*S. aureus* (G+), *V. splendidus* (G−), *V. harveyi* (G−), *V. anguillarum* (G−), *E. cloacae* (G−), *A. hydrophila* (G−)	LPS, glucan	[55]
	VpCTL	*S. aureus* (G+), *Escherichia. coli* (G−), *V. parahaemolyticus* (G−), *V. harveyi* (G−), *P. pastoris* (fungi)	1.75-fold increasing	Increasing up to 45% after 6 h and up to 90% after 24 h.	N/D	LPS, PGN, glucan, zymosan	[96]
*S. farreri*	CFLec-1	*E. coli* (G−)	N/D	N/D	*M. luteus* (G+), *E. coli* (G−)	N/D	[58]
	CfLec-1	N/D	2.5-fold increasing	Increasing up to 62% after 6 h and up to 75.6% after 24 h.	N/D	LPS, PGN, mannan	[59]
	CfLec-2	N/D	N/D	Increasing up to 76% after 6 h.	N/D	mannan, LPS, PGN, zymosan	[60]
	CfLec-3	*S. aureus* (G+), *E. coli* (G−), *V. anguillarum* (G−), *Pseudomonas stutzeri* (G−), *P. pastoris* (fungi), *Y. lypolytica* (fungi)	2.89-fold increasing	Increasing up to 87.3% after 6 h (against *E. coli*)	N/D	LPS, PGN, β-glucan, mannan	[62]
							[61]
	CfLec-4	*S. aureus* (G+), *M. luteus* (G+), *E. coli* (G−), *V. anguillarum* (G−), *P. pastoris* (fungi)	2.9-fold increasing	N/D	N/D	LPS, PGN, mannan, glucan	[63]
		CRD1, CRD2: *S. aureus* (G+), *V. anguillarum* (G−). CRD3: *S. aureus* (G+), *M. luteus* (G+), *E. coli* (G−), *V. anguillarum* (G−), *P. pastoris* (fungi), *Y. lypolytica* (fungi). CRD4: *S. aureus* (G+)	CRD1: 1.59-fold increasing. CRD2: 1.22-fold increasing. CRD3: 1.8-fold increasing. CRD4: 1.4-fold increasing	N/D	N/D	CRD1, CRD2: LPS, mannan. CRD3, CRD4: LPS, PGN, mannan, glucan	[97]
	Cflec-5	*P. pastoris* (fungi)	N/D	N/D	N/D	glucan, LPS	[65]
*S. constricta*	ScCL	*S. aureus* (G+), *V. harveyi* (G−)	N/D	N/D	N/D	LPS, lipoteichoic acid	[66]
	ScCTL	*M. luteus* (G+), *E. coli* (G−), *V. harveyi* (G−), *V. anguillarum* (G−), *V. parahaemolyticus* (G−)	N/D	N/D	N/D	N/D	[67]
	ScCTL-1	*S. aureus* (G+), *V. anguillarum* (G−)	1.3-fold increasing	N/D	N/D	N/D	[68]
	ScCTL-2	*E. coli* (G−), *V. anguillarum* (G−), *V. parahaemolyticus* (G−)	N/D	N/D	N/D	N/D	[69]
*T. granosa*	TgCTL-1	*B. subtilis* (G+), *S. aureus* (G+), *E. coli* (G−), *A. hydrophila* (G−), *V. parahaemolyticus* (G−)	1.67-fold increasing	N/D	N/D	N/D	[71]

(G+)—Gram-positive bacteria; (G−)—Gram-negative bacteria; N/D—not determined.

**Table 4:** Systematic summary of conserved motifs in the CRD domains of bivalve CTLs and their carbohydrate specificity.

Species	C-Type Lectin	Carbohydrate Binding Motif	Coordinating Motif	Carbohydrates and Glycoconjugates (Inhibition Concentration)	Ca^2+^-Dependent/Ca^2+^-Independent	Ref.
*A. irradians*	AiCTL1	QPD	WRD *	N/D	N/D	[32]
	AiLec	EPD	WND	N/D	N/D	[37]
	AiCTL-6	EPD	WSD	N/D	Yes	[34]
	AiCTL-7	EPD	WSD	D-mannose (0.035 mM), D-galactose (0.035 mM)	Yes	[35]
	AiCTL5	QPN	WND	N/D	Yes	[92]
	AiCTL-3	EPN	WND	D-mannose (0.035 mM), D-galactose (0.035 mM)	N/D	[33]
	AiCTL-9	CRD1-YPT, CRD2-EPD, CRD3-EPN, CRD4-QPN	CRD1-FQN, CRD2-FSD, CRD3-YND, CRD4-YMV	N/D	Yes	[36]
*C. virginica*	CvML	YPD	WID	N/D	N/D	[38]
	CvML3912	QPN	WGD *	N/D	N/D	[73]
*M. gigas*	CgCLec-1	YPD	WID	N/D	N/D	[39]
	CgClec-2	EPN	WFD	N/D	Yes	[40]
	CgCLec-4	QPE *	WHD	N/D	Yes	[42]
	CgCLec-5	QYE *	LTS *	N/D	Yes	[42]
	CgCLec-3	DIN*	-	mannose (0.45 mM)	Yes (despite the absence of a coordinating motif)	[41]
	CgCLec-HTM	QPS	WHD	N/D	N/D	[95]
	CgLec-4E	EPA *	WHD	N/D	Yes	[45]
	CgCLEC-TM2	EFG *	FVN *	N/D	Yes	[43]
*M. meretrix*	MmCTL4	QPN	WSD	D-xylose (25 mM), D-mannose (100 mM), D-lactose (100 mM), maltose (100 mM)	Yes	[46]
	MmCTL5	QPS	WND	D-xylose (100 mM), D-mannose (200 mM), D-galactose (200 mM), D-lactose (200 mM), D-glucose (200 mM), maltose (200 mM)	Yes	[47]
*M. edulis*	M3	QMI *	FHW *	Asialofetuin	Yes	[109]
	M6	QPD	FLD	Asialofetuin	Yes	[109]
	M7	QPD	FLD	Asialofetuin	Yes	[109]
	MeML	QPS	WND	N/D	N/D	[50]
*R. philippinarum*	MCL3	QPD	WND	mucin type II: Galβ1,3GalNAcβ1,6GlcNAc-Ser/Thr (0.67 mM), raffinose (27 mM), N-acetylgalactosamine (27 mM)	Yes	[52]
	RpCTL	EPN	WND	N/D	N/D	[53]
	VpClec-1	QPD	WLD *	N/D	Yes	[54]
	VpClec-2	EPN	ISG *	N/D	Yes	[54]
	VpClec-3	EPN	WND	N/D	Yes	[55]
	VpClec-4	QPN	WVD *	N/D	Yes	[55]
	VpCTL	EPN	WND	N/D	Yes	[56]
*S. purpurata*	SPL-1	RPD * (A-chain), KPD * (B-chain)	WND	N-acetylglucosamine (5 mM), N-acetylgalactosamine (25 mM)	Ca^2+^ cations are not strictly required	[110]
	SPL-2	KPD * (B-chain)	WND	N-acetylglucosamine (5 mM), N-acetylgalactosamine (25 mM)	Ca^2+^ cations are not strictly required *	[110]
*S. farreri*	Cflec-5	EPN	WND	D-mannose (200 mM)	N/D	[65]
	CfLec-2	EPD	WFD	D-mannose (200 mM)	Yes	[98]
	CfLec-1	EPD	MLD *	N/D	Yes	[58]
	CfLec-4	CRD1-EPD, CRD2-EPN, CRD3-EPN, CRD4-EPN	CRD1-LSD *, CRD2-FAD *, CRD3-LND *, CRD4-YND	N/D	Yes	[64]
	CfLec-3	CRD1-YPT, CRD2-EPD, CRD3-EPN	CRD1-FQN, CRD2-FSD, CRD3-YND	D-mannose (200 mM)	Yes	[61]
*S. constricta*	ScCTL-2	-	WHD	N/D	No (Ca^2+^-independent)	[69]
	ScCTL	VPD *	WND	N/D	Yes	[67]
	ScCL	EPN	WHD	N/D	Yes	[66]
	ScCTL-1	CRD1-HPD *, CRD2-EPD, CRD3-EPN, CRD4-MPT *	CRD1-VSD *, CRD2-FLD, CRD3-FND *, CRD4-YIN *	N/D	Yes	[68]
*C. orbicularis*	Codakine	EPN	WND	Man3GlcNAc2 (carbohydrate motif), D-mannose (25 mM), L-fucose (25 mM), D-glucose (100 mM), N-acetylglucosamine (100 mM)	Yes	[111]
*S. grandis*	SgCTL-1	EPN	WHD	N/D	N/D	[70]
*M. yessoensis*	MyCLF	EPN	WDD	N/D	N/D	[49]
*M. crassicostata*	Cnlec-1	CRD1-YPT, CRD2-EPD, CRD3-EPN, CRD4-EPN	CRD1-FQN, CRD2-FSD, CRD3-YBD, CRD4-YMV	N/D	N/D	[48]
*P. fucata*	PmCTL-1	QPN	WID	N/D	N/D	[51]
*T. granosa*	TgCTL-1	QPN	WDD	N/D	Yes	[71]
*G. yessoensis*	GYL	EPN	WND	Galβ1-4GlcNAcβ (carbohydrate motif), L-fucose (0.17 mM), PSM (0.033 mg/mL), asialo-PSM (0.008 mg/mL), fetuin (0.008 mg/mL), asialofetuin (0.004 mg/mL), thyroglobulin (0.004 mg/mL), ovalbumin (0.025 mg/mL)	Yes	[94,112]

*—unique motifs identified by multiple sequence alignment; N/D—not determined.

## Data Availability

No new data were created or analyzed in this study. Data sharing is not applicable to this article.

## Abbreviations

The following abbreviations are used in this manuscript:

CRD	carbohydrate-recognition domain
CTL	C-type lectin
CTLD	C-type lectin-like domain
IHC	immunohistochemistry
ISH	in situ hybridization
LPS	lipopolysaccharide
PAMP	pathogen-associated molecular pattern
PGN	peptidoglycan
PIM	percent identity matrix
PRR	pattern recognition receptor
PSM	porcine submaxillary mucin
qPCR	quantitative real-time polymerase chain reaction
